# An aqueous extract of the brown alga *Eisenia bicyclis* extends lifespan in a sex-specific manner by interfering with the Tor-FoxO axis

**DOI:** 10.18632/aging.204218

**Published:** 2022-08-16

**Authors:** Navid Tahanzadeh, Mirjam Knop, Yvonne Seidler, Sebastian Dirndorfer, Kai Lürsen, Iris Bruchhaus, Roman Lang, Gerald Rimbach, Thomas Roeder

**Affiliations:** 1 Kiel University, Department Molecular Physiology, Zoology, Kiel, Germany; 2 Kiel University, Institute of Human Nutrition and Food Science, Kiel, Germany; 3 Leibniz Institute for Food Systems Biology, TU Munich, Munich, Germany; 4 Bernhard-Nocht-Institute for Tropical Medicine, Department Parasitology, Hamburg, Germany; 5 DZL, German Center for Lung Research, ARCN, Airway Research Center North, Kiel, Germany

**Keywords:** lifespan, alga, Tor, sex-specific

## Abstract

Food has a decisive influence on our health, to the extent where even lifespan can be directly affected by it. In the present work, we have examined the effects of an aqueous extract of the marine brown alga Eisenia bicyclis in terms of its potential to extend lifespan. For this purpose, we used the fruit fly *Drosophila* melanogaster as a model. The experiments showed that small amounts of Eisenia extract can extend lifespan by up to 40%. This effect is not only related to the median but also to the maximum lifespan. Interestingly, this life-extending effect is sex-specific, i.e. it occurs exclusively in females. Even under stressful nutritional conditions such as a high sugar diet, this effect is detectable. Mechanistic studies showed that this life-prolonging effect depends on a functional Tor and a functional FoxO signaling pathway. It can be concluded that components of the Eisenia extract prolong lifespan by interacting with the Tor-FoxO axis. This study may serve to stimulate further investigations, which on the one hand show such a life-prolonging effect also in other organisms and on the other hand identify the substances responsible for this effect. Finally, it may also encourage the increased use of arame as a health-promoting food supplement.

## INTRODUCTION

The composition of the diet has a major impact on our lives. This includes not only several different health aspects, but in fact even the lifespan. In this context, different dietary compositions can convey either positive or negative effects. These effects usually concern the macronutrients proteins, fat and carbohydrates [1, 2]. Micronutrients, on the other hand, can also mediate positive effects on health, but these effects are much less well understood so far. Extracts or isolated components, especially from plants, algae and fungi, serve as sources for these positive effects, which are achieved through a modified composition of micronutrients. It can be assumed that the life-prolonging effects of these micronutrients in the plant-fungal and algal extracts are due to a pharmacological action. This pharmacological effect could, ideally, mimic the effects of a macronutrient intervention such as a caloric restriction (CR) or a dietary restriction (DR) [3]. Exemplary for such an effect is rapamycin, a macrolide first isolated from the fungus *Streptomyces hygroscopicus* [4]. Rapamycin has shown its potential as a lifespan prolonging agent in several studies using different models [5–7], but its use as a regularly taken medication in humans, with the aim of prolonging the lifespan is under intense discussion due to the observed and anticipated side effects [8]. Besides rapamycin and rapamycin-related compounds, alpha-ketoglutarate is one of the very few compounds that showed its potential as a lifespan prolonging agent. Administration of alpha-ketoglutarate, an intermediate of the TCA cycle has been shown to increase life- and health span in a sex-dependent manner by interfering with Tor signaling and by reducing inflammation [9–12]. What these two substances have in common is that they interact with the Tor signaling pathway, suggesting that interaction with this pathway may be a general property of life-prolonging substances [13].

To expand the range of substances that show a health and lifespan promoting effect, extracts of plants and algae seem to be excellent. This insight is based, among other things, on the observation that certain populations that consume diets with a large proportion of certain plants and algae are markedly healthy and long-lived. Southeast Asian groups, for example, who consume a lot of marine products, are worth mentioning [14, 15]. To exploit this important resource, we conducted a broad-based screen in which we examined a larger number of different plant and algal extracts with respect to their life-prolonging effects. For this purpose, we used the fruit fly *Drosophila* as a model, since only it and the nematode *C. elegans* can fulfil the requirements for such a screen [5, 16]. Although the first use of the fly in studies focusing on the effects of interventions on lifespan dates almost 70 years back [17], the majority of studies employed *C. elegans*, as it is easier to use in high-throughput formats [18]. Nevertheless, the fruit fly, similarly as other insects such as the red flour beetle *Tribolium* [19], offers several advantages compared with *C. elegans* comprising an organ composition and nutritional physiology that shares considerable similarities with those of humans [20, 21]. Based on these screening systems, a series of plant and algal extracts have been identified that hold the potential to increase lifespan, but unfortunately most studies lack any mechanistic analysis. A recent study showed that an aqueous extracts of furbelow, a brown alga (*Saccorhiza polyschides*) can induce a robust prolongation of lifespan in *Drosophila*. This was seen under control conditions but even more pronounced under different stress conditions [22]. The identical extract was able to mediate comparable effects in mice, although here the focus was more on health-promoting effects [23]. Mechanistically, the effects on lifespan could be attributed to an interference with the Tor signaling pathway [22].

In the present study, we investigated the effects of an aqueous extract of the brown alga Arame (*Eisenia bicyclis*) for its life-prolonging potential. Here, a sex-specific positive effect on lifespan was shown that was also seen under nutrient and other stress conditions. Moreover, we identified that this effect was Tor-dependent. Thus, we could show that this alga, which is used for a long time as a food supplement in Japan and [24, 25], has the potential to increase health- and lifespan if used as a food supplement.

## RESULTS

### *Eisenia bicylis* extracts extend lifespan of *Drosophila melanogaster* in a sex-dependent manner

**Figure 1:**
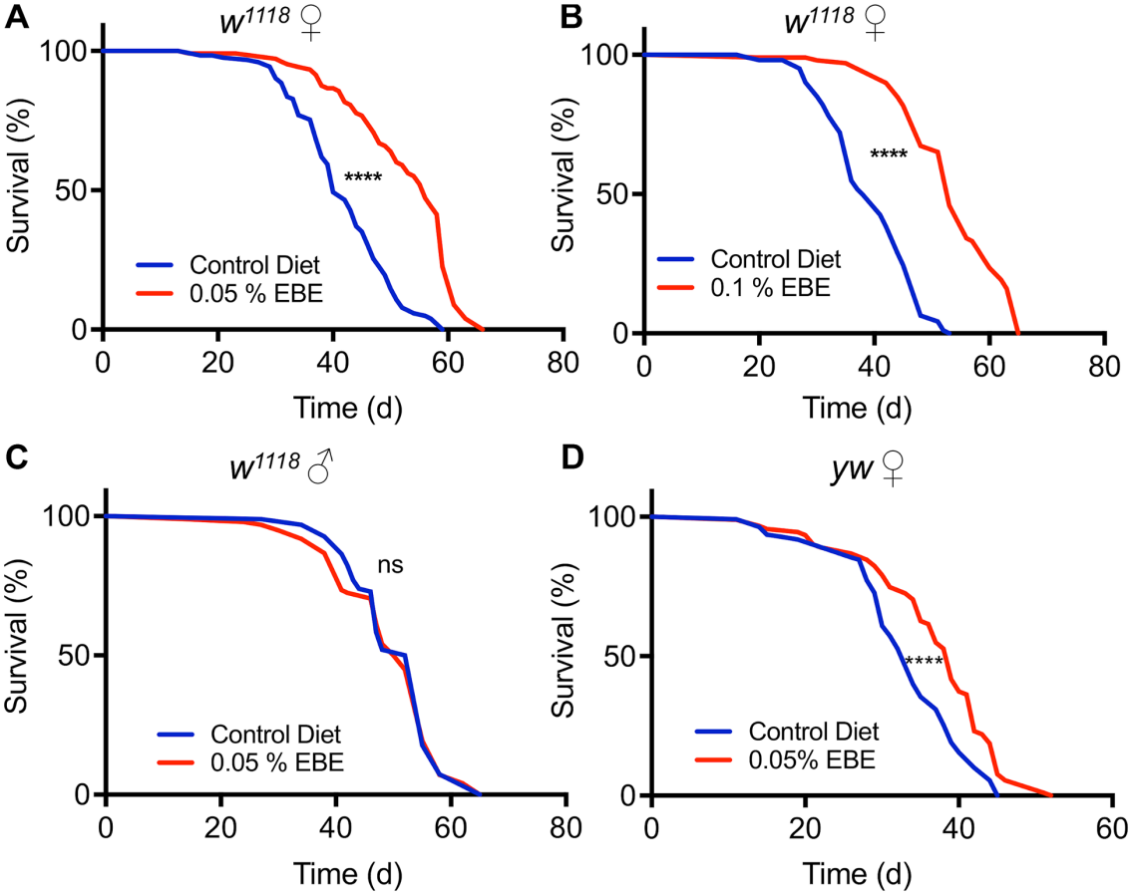
**Application of *Eisenia bicyclis* extract (EBE) enhances lifespan in female *Drosophila*.** Lifelong application of 0.05% EBE (red) to adult female *Drosophila* (w^1118^) was compared to control flies of the same genotype (blue). The proportion of surviving animals is displayed against time (**A**). In (**B**), 0.1 % EBE was used under otherwise identical conditions. A similar analysis was performed with w^1118^ males and 0.05% EBE was applied (red) and compared with controls (blue) (**C**). Female flies of the yw strain were confronted with 0.05 EBE (red and compared to matching controls (blue) (**D**). (n > 100 per condition). Statistical analyses were done using a log-rank test. Ns means not significant, *** means p< 0.005, **** means p<0.001.

An aqueous extract of the brown alga Arame (*E. bicyclis)* was identified during a larger screen of plant and algal extracts for their life extending potential using fruit flies as a model system [22, 26]. To further characterize and to verify this lifespan prolonging effect, we used two different concentrations (0.1% and 0.05%) of this extract and measured lifespan in cohorts of mated females of the *Drosophila melanogaster w^1118^* strain. Animals subjected to 0.05 % of the *E. bicyclis* extract (EBE) showed a lifespan prolongation (Figure 1A), with an increase in median lifespan of about 40 % (p<0.0001). Application of the extract at a higher concentration (0.1%) showed the absence of adverse effects and that this higher concentration increased the median lifespan to a very similar extent (39.5 %, p<0.0001). The increases in lifespan were not only seen for the median lifespan, but also for the maximal lifespans (10% of animals with highest lifespans) under both conditions (56 d to 63 d and 51 d to 65 d, respectively, p<0.0001 each, see also Supplementary Table 1). We also tested cohorts of mated males, but we did not see any lifespan-extending effect by application of the *Eisenia* extract (Figure 1C). To exclude that the observed effects on lifespan are only strain-specific, we tested a second *Drosophila* strain, namely *y^1^w^1118^*. Here, we could also show a robust lifespan prolongation induced by the EBE (Figure 1C; p<0.0001). Again, also the maximum lifespans were increased (45d to 48.5 d, p=0.0006).

**Figure 2:**
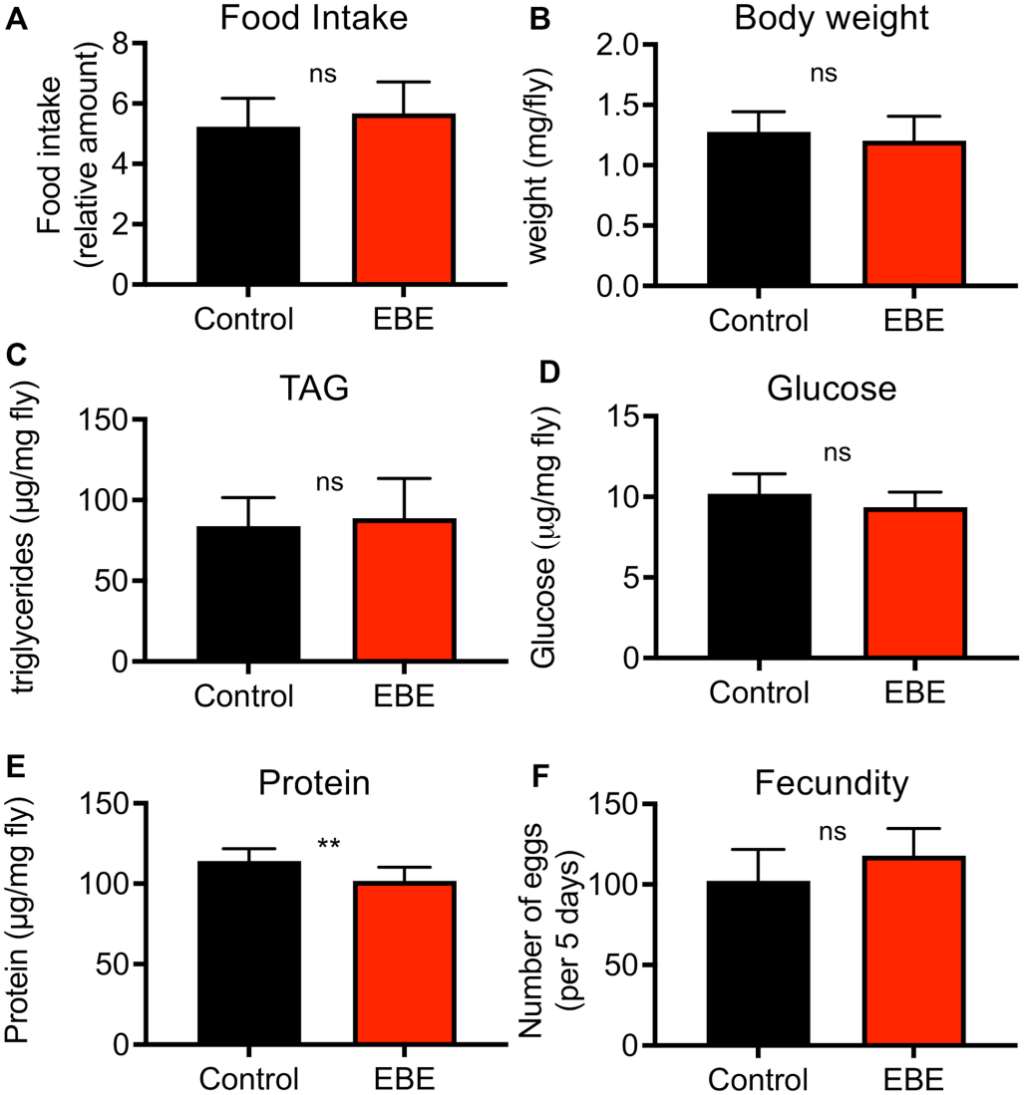
**Effects of *Eisenia bicyclis* extract of physiological parameters of female *Drosophila*.** Application of EBE to female adult flies did not change the intake of nutrients over a 24 h period (**A**). The body weight was also not changed in response to EBE (**B**). The effects on body fat (**C**), on body glucose (**D**) and body protein (**E**) were quantified. Fecundity was quantified under both conditions (**F**). N≥10, mean values ± S.E.M. are given. Statistical analyses were performed with unpaired t-tests. Ns means not significant, ** means p< 0.01.

To exclude indirect effects caused by reduced uptake and therewith leading to an induced caloric restriction with its lifespan prolonging effects, we quantified the nutritional intake using the consumption-excretion assay. Here, no statistically significant differences in food consumption during a 24 h period could be observed (Figure 2A, p=0.3015). Moreover, we treated the flies with 0.05 % EBE and analyzed the body composition. Therefore, we analyzed parameters such as body weight, protein content, triacylglycerol levels, and glucose content. Regarding the body weight, the experimental groups did not show any difference (p=0.1606) (Figure 2B). We examined lipid storage and carbohydrate content by measuring triglyceride (TAG) and glucose levels, respectively and have related that to body weight, since this is the only independent reference variable. TAG levels and glucose content of whole flies fed with the extract also showed no significant changes (p=0.1023 and p =0.6134 respectively) (Figure 2C, 2D). Only with respect to the body protein content of EBE-treated flies, a significant but mild reduction compared to the control group was observed (p=0.0033) (Figure 2E). To demonstrate that the observed lifespan extension does not compromise other measures of health, a fecundity test was performed. It revealed that algal treated flies produced similar amounts of eggs as the control group (p=0.1051) (Figure 2F).

### Phenotypic analysis of the effects of EBE treatment

**Figure 3:**
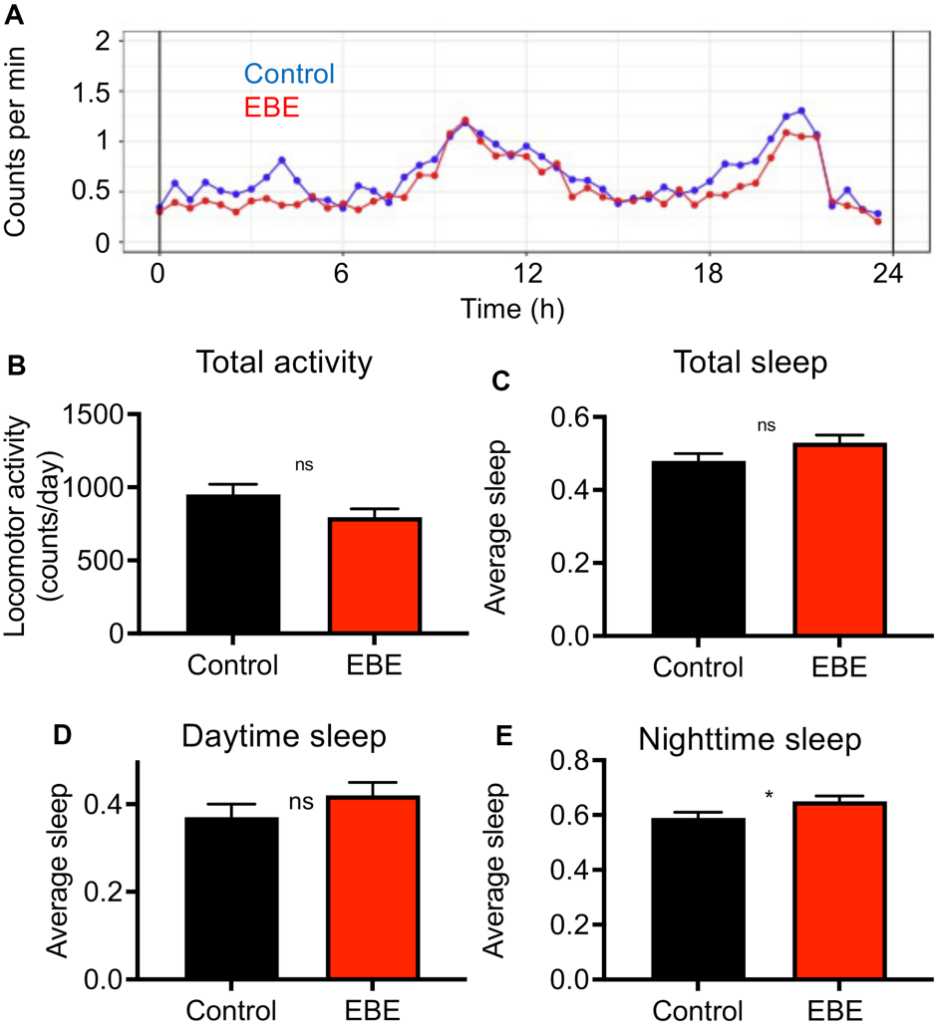
**Influence of *Eisenia bicyclis* extracts on activity and sleep of adult female flies.** Activity traces derived from a DAM-based analysis (**A**) of mated female flies in a 24h period. The total mean activity in a 24h period (**B**), the accumulated total sleep time (**C**), and the accumulated daytime sleep time (**D**) and the accumulated nighttime sleep time (**E**). N≥10, mean values ± S.E.M. are given (**B**–**E**). Average sleep amounts are stated as a fraction of 1. Statistical analyses were performed with unpaired t-tests. Ns means not significant, * means p< 0.05.

We analyzed the effect of EBE intake on additional phenotypic characteristics. In doing so, we subjected the influences on the activity behavior to a special analysis (Figure 3). The general activity pattern hardly differs between control and EBE-treated animals (Figure 3A), the same is true for the cumulative activity over a 24 h period (Figure 3B). We also analyzed sleep amounts and sleep patterns, because sleep duration has been associated with lifespan [27]. A more detailed analysis of sleep behavior revealed slightly, although not significantly, increased sleep episodes after EBE treatment over a 24 h period (Figure 3C). However, further breakdown into daytime sleep activity (Figure 3D) and nighttime sleep activity (Figure 3E) revealed that the amount of nighttime sleep was significantly increased.

### *E. bicyclis* extracts improve resistance to some stress conditions

**Figure 4:**
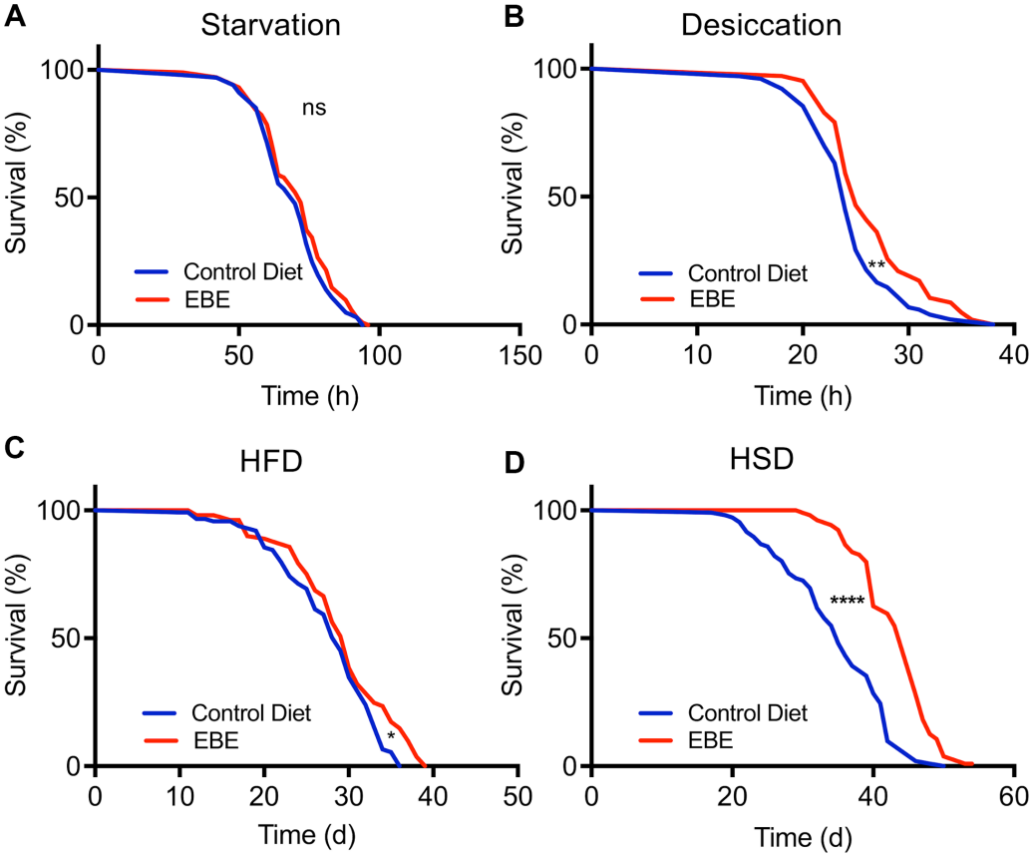
**Influence of *Eisenia bicyclis* extracts of lifespan in response to different stressors.** Lifespan of w^1118^ females in response to starvation (**A**) and desiccation (**B**) in control flies and those subjected to 0.05 % EBE. Lifespan of W1118 females on a high-fat diet (**C**) and a high sugar diet (**D**) (n > 100 per condition). Statistical analyses were done using a log-rank test. ns means not significant, * means p<0.05, ** means p< 0.01, **** means p<0.001.

We next analyzed if addition of the EBE changed the lifespan in response to stressful conditions. We first assessed resistance to starvation by placing control and EBE-treated flies on agar only, to prevent energy intake, while sufficient water is supplied. Under these conditions, the median lifespan was 72 h and 70 h for the EBE treated and the control group, respectively. However, the difference was not statistically significant (p=0.1997) (Figure 4A and Supplementary Table 1). In contrast, adding EBE to the flies’ food increased the resistance to desiccation. The median lifespan for the control group was 24 h, whereas this increased to 25 h in EBE-treated flies (p=0.0027) (Figure 4B). Subsequently, we tested the effects of the major nutritional stressors, namely high-fat, and high sugar diets. Flies treated with 0.05 % EBE had a slight, but significant lifespan extension on a high-fat diet (HFD). The median lifespans were 29 and 30 days for the control group and the EBE treated groups, respectively (p=0.0140) (Figure 4C). Moreover, we reared flies on food containing 30 % sugar and asked whether the administration of EBE reduced the deleterious effects of the high sugar diet. Here, we found that the EBE addition led to an increase in median lifespan by about 30% from 35 days under control conditions to 46 days in response to EBE application (Figure 4D; p-value <0.0001). The maximum lifespans also showed a significant increase (from 46 in controls to 50 after EBE, p<0.0001).

**Figure 5:**
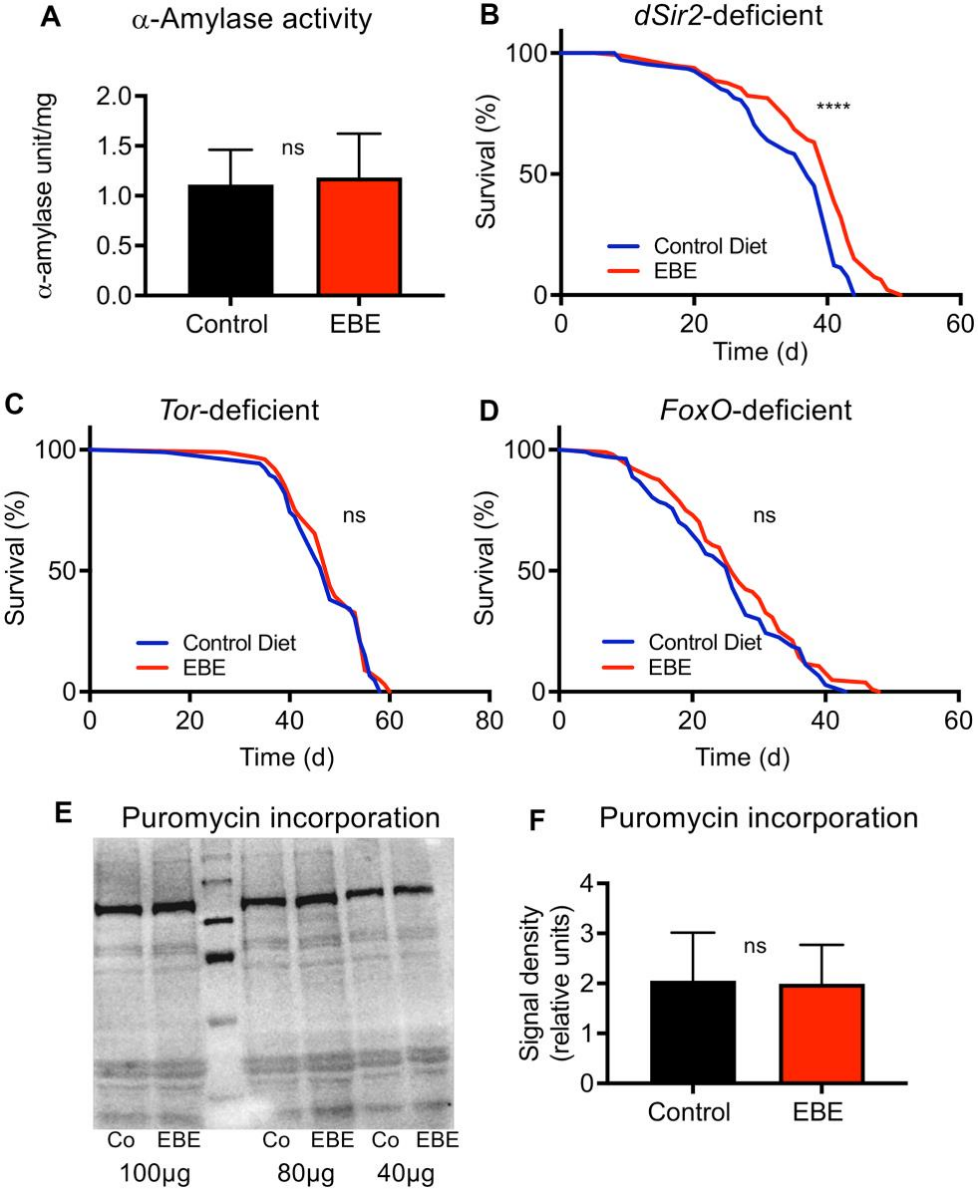
**Mode of action of the *Eisenia bicyclis* extract induced lifespan prolongation.** The alpha-Amylase activity of control flies and EBE treated ones (**A**) are displayed. Lifespan analyses of control and EBE-treated female flies of the genotypes: dSir2-deficient (**B**), TOR-deficient (**C**), and FoxO-deficient (**D**). Western blot analysis of samples from control flies (Co) and EBE-treated ones (EBE) of puromycin treated flies (**E**) and the quantification of bad intensities (**F**). lifespan did not increase on concentrated medium containing 0.05 % EBE, (n > 100 per condition). Protein synthesis of flies treated with 0.05 % EBE was not affected (**E**, **F**). N≥10, mean values ± S.E.M. are given (**A**, **F**). Statistical analyses were performed with unpaired t-tests. ns means not significant. (**B**–**D**, n > 100 per condition). Statistical analyses were done using a log-rank test. ns means not significant, **** means p<0.001.

Due to the effects of EBE on HSD mentioned earlier, we quantified the body composition of flies reared on a HSD (Figure 5). The body weight, total TAG levels and whole-body protein did not change under these conditions (Supplementary Figure 1). Only the body glucose level in glucose level of EBE treated flies was significantly lower if compared to the control group (p= 0.0443) (Supplementary Figure 1).

### Mode of action of the lifespan prolonging effects of EBE

To elucidate potential mechanisms by which the extract extends the lifespan of flies, we tested the alpha-amylase activity in flies in response to EBE treatment as this has been shown to be modulated by *Eisenia*-derived phlorotannins [28]. Here, no differences in the alpha-amylase activities between control animals and those subjected to EBE were seen (Figure 5A). We then assessed which signaling pathways are responsible for the lifespan-prolonging effects of an EBE treatment. We first evaluated Sir2-dependent processes since activation of the Sir2 pathway was clearly identified as life-prolonging in *Drosophila* [29]. By analyzing *sir2*-deficient flies, we could show that Sir2 is obviously not the relevant EBE target, as the corresponding flies nevertheless showed EBE-induced lifespan extension (Figure 5B). Nevertheless, the reduced increase in lifespan could also be interpreted in a way that Sir2 participates in the life-prolonging effect. Furthermore, we analyzed the Tor signaling pathway and tested Tor-deficient animals for this purpose. Here, we could show that EBE application did not show any lifespan-prolonging effect in these animals, proving that the Tor signaling pathway is closely linked to the EBE-induced effect (Figure 5C). Since FoxO signaling is closely linked to lifespan on the one hand and can act downstream of Tor on the other [30], we also checked this factor and analyzed *foxo*-deficient animals. Again, we could not detect any EBE-induced positive effect on lifespan (Figure 5D). This means that FoxO is also required for EBE-mediated effects on lifespan. Since Tor-signaling is also more important for proteostasis, we checked protein synthesis using the puromycin assay (Figure 5E, 5F). Here, we could not determine any effect of EBE treatment on new protein formation (Figure 5E, 5F).

### Qualitative HPLC-MS analyses of aqueous extracts

**Figure 6:**
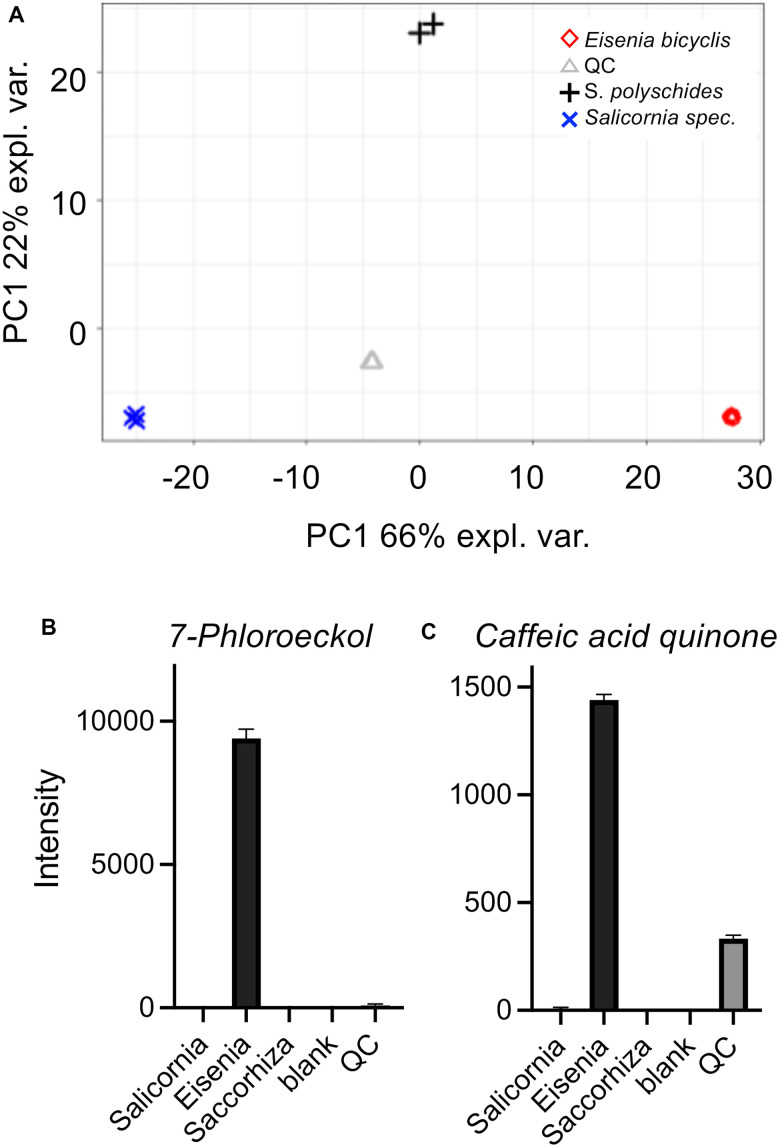
**Untargeted HPLC-MS analysis of algae extracts.** Principal component analysis (PCA) (**A**) of algae extracts measured using the ESI+ and ESI- acquisition mode. Underlying peak areas are log10 transformed and pareto-scaled. (**B**, **C**) LC-ToF-MS intensities of 7-phloroeckol (**B**) and caffeic acid quinone (**C**). Intensities are displayed as the mean of triplicate injections (duplicate injections for Saccorhiza).

We performed a comparative HPLC-MS study to characterize the algae material and find the very first clues about the possible substances responsible for the life-prolonging effects. For this purpose, we compared aqueous extracts of three marine plants/algae with different properties to focus especially on those substances that are specifically found in EBE. The extracts in question were those of *Eisenia bicyclis*, of the brown alga *Saccorhiza polyschides*, and of the picklegrass (*Salicornia spec.*). The EBE is in the center of the current study and the *Saccorhiza polyschides* extract has been characterized in detail to exert life- and/or health-span extension in flies and mice [22, 23]. The *Salicornia* extract on the other hand is of marine origin, but not an algae and serves as a control. The extracts were separated by chromatography on C18, compounds detected by Time-of-Flight mass spectrometry (ToF-MS) and identified based on library search (cf. methods and Supplementary Material). The MS analysis gave relative concentration differences between the samples. Principal component analysis (PCA) showed that the compositions of the extracts were clearly distinguishable (Figure 6A). By performing a partial least-squares discriminant analysis (PLS-DA) (Supplementary Figure 2), the top 100 features which are higher abundant in *Eisenia bicyclis* samples could be determined and sorted by their importance (Supplementary Table 5). Moreover, we show differentially present metabolites as a heatmap (Supplementary Figure 3). Of these 100 selected metabolites with differential occurrence in the different samples, we focused on those metabolites that are specifically found in EBE and for which information on life-prolonging potential is already available. In particular, 2 substances stood out, 7-phloroeckol (Figure 6B) and caffeic acid quinone (Figure 6C). In the case of 7-phloroeckol, however, a differentiation to the isoflavonoid C-glyoside UNPD147636 is not possible due to identical mass numbers. It should be noted, however, that 7-phloroeckol is one of the "lead substances" of the brown alga of the genus *Eisenia*, whereas isoflavonoid C-glyosides are hardly known in algae.

## DISCUSSION

In the study presented here, we followed the goal of identifying algae extracts that exert a life-prolonging effect and tried to explain this effect mechanistically. The long-term goal, which is of course in the background, was to recommend such extracts as a food supplement for human use. We were able to show that an aqueous extract of the brown alga Arame (*Eisenia bicyclis*) produced a marked prolongation of the lifespan, which accounted for almost 40 % of the median lifespan. This extension affected not only the median but also the maximum lifespan. Thus, this study joins others that have already shown a positive effect of algae and plant extracts in the *Drosophila* model [22, 31–34]. To ensure that this is a true pharmacological effect and not a confounder based on reduced food intake, we investigated whether the extract affects food intake. We were able to exclude this and thus rule out that aversive effects of an extract on food intake induce a CR caused by the altered behavior. Unfortunately, such controls are rarely investigated in other studies, which severely limits their power. In addition, we were able to independently demonstrate life extension in a second *Drosophila* strain. The observation that different wild-type strains show different lifespans has already been demonstrated but does not interfere with interventions that change lifespan [35]. This clearly indicates that the observed life-prolonging effects should generally apply to *Drosophila melanogaster*.

Interestingly, the rather substantial lifespan prolonging effect induced by EBE was sex specific. This means that only females showed this positive effect and males were not affected. This is different from a recent study employing extracts from another brown alga, namely of the furbelow *Saccorhiza polyschides,* where both sexes showed similar lifespan extensions, although the degree of lifespan-extension was substantially smaller [22]. Such sex-specific effects have been shown more frequently. It seems to be a phenomenon that is particularly significant in aging studies. In most cases, females are more susceptible to interventions and show larger effects [10, 36]. This is also true for interventions such as CR (Regan et al. 2016). Metabolic processes clearly differ between both sexes in *Drosophila* [37, 38], and it was shown that female flies are far more responsive to dietary interventions such as dietary restriction than males are [39]. This implies that there is a general bias in responsiveness towards females in nutritional interventions.

Regarding the underlying mechanism, we clearly identified the Tor-FoxO axis as the site of action of EBE effects. In contrast, the Sir2 pathway and associated signaling pathways do not seem to be relevant for the observed life-prolonging effect. The distinctly complex Tor signaling pathway, in which Tor is the central control unit, appears to be of general importance for age-related processes. This is illustrated by the fact that the best characterized life-prolonging substance to date, rapamycin, was the eponym for this protein. Tor signaling takes a central role because it permanently determines the nutritional state of the cell, in particular the protein balance of the cell, and uses this information to adjust cell metabolism accordingly. For this reason, the Tor signaling pathway is also considered the central target of a CR or DR intervention. Consequently, substances or extracts that interfere with the Tor signaling pathway and that exert a life-prolonging effect can also be referred to as DR mimetics. Inhibition of the Tor pathway to prolong lifespan has been demonstrated in a variety of systems [40–42]. Therefore, it can be assumed that one or more components from the EBE specifically interfere with the Tor signaling pathway and thus exert the life-prolonging effect. Interestingly, we were also able to show that, in addition to the Tor signaling pathway, FoxO and thus FoxO-dependent processes are also necessary for the life-prolonging effects of EBE treatment. This can be explained relatively simply by the fact that there is a Tor-FoxO axis that is precisely responsible for these processes. The functionality of such an axis has already been shown in other systems [30, 43, 44]. Accordingly, it can be assumed that this is also a functional Tor-FoxO axis, in which an EBE component leads to inhibition of the Tor signaling pathway and this effect is translated via FoxO into an extension of the lifespan. Nevertheless, it remains obscure, why males did not react although their Tor/FoxO axis appears to be very similar compared to those of females.

Another aspect that could be of importance in this context is the observed slight prolongation of night sleep. This is relevant because there is a correlation between sleep duration and sleep patterns as well as lifespan [45–47]. An improvement in night sleep could therefore be associated with an increase in lifespan.

It can be concluded that EBE impart lifespan prolonging effects. It has already been described that health-promoting effects can be attributed to this alga. Here, especially inhibition of inflammatory responses [48, 49], cancer development [50, 51], and inhibition of ROS-mediated damages [25, 52, 53] had been characterized. The bases identified and anticipated for these described effects differed fundamentally from each other and could be attributed to different substance classes. Inspired by these studies, several different substances, or classes of substances, from *Eisenia bicylis* were investigated in detail. Here, a special focus was on phlorotannins that directly interfered with enzyme activities such as α-glucosidase, alpha-amylase, or pancreatic lipase [28, 54].

Moreover, these phlorotannins from *Eisenia bicylis* have also been shown to protect against ROS stress, reduce inflammatory responses, and to show antibacterial activities [48, 49, 55–57]. Among the phlorotannins, different eckol derivatives are the most often characterized representatives [58]. 7-Phloroeckol, the substance that we identified as one of those that are highly specific for Eisenia extracts, is one of them. Recently, it has been associated with antidiabetic effects and with a pronounced inhibition of alpha-amylase activities. In our study, we were unable to show inhibition of alpha-amylase activities by EBE, which is not surprising as the effective inhibitory concentration of these eckols is in the range of 1mM [59, 60], a concentration that will never be reached in our assay, where the unfractionated extract accounts for less than 0.1% of the total food. Thus, 7-phloroeckol is a candidate compound that might have additional effects acting at much lower concentrations Besides the phlorotannins, fucosterols have been shown to be associated with relevant biological activities [48, 53, 61]. One last compound of interest is caffeic acid quinone, which is an oxidized product of caffeic acid [62], with caffeic acid being one compound with the proven ability to prolong lifespan [63]. In addition to the different compounds of algae extracts that elicit biological functions, a different mode of action has also been presented, namely, an epigenetic effect that can modulate inflammatory responses [64].

Taken together, it can be concluded that we could show with this study that a simple aqueous extract of *Eisenia bicylis* can substantially prolong the lifespan of female *Drosophila*. This sex-specific effect is seen in different *Drosophila* strains and under diverse stressors. The extract components responsible for this effect interact very specifically with the Tor signaling pathway, which in turn uses the FoxO signaling pathway to mediate these life-extending effects. Because of the other health-promoting effects already described for *Eisenia bicyclis* extracts, such extracts should also be excellent supplements to human diets. This is insofar relevant as it exerts its positive effects at concentration of less than 0.1% of the total ingested diet, making the EBE a very promising food supplement that could be used with only a few grams per day. On the other hand, this study may serve to identify the active components from the extract in subsequent studies. We propose few candidate compounds that might be responsible for mediating the observed effects with 7-Phloroeckol being the most promising one for future research that also might use mice to test if lifespan prolongation is also seen there.

## CONCLUSIONS

In summary, the results of this project state that an aqueous extract of the brown alga *Eisenia bicylis* substantially extends both the mean and maximum lifespan of different strains of *Drosophila melanogaster*. This effect is clearly sex-specific, being observed only in females. Extract does not affect food intake or alter significant physiological parameters. Even under stress conditions, this supplement conferred a very clear advantage. The analysis of the underlying mechanism revealed that the life-prolonging effect relies unambiguously on the Tor/FoxO axis and is not expressed in the absence of these signaling pathways. We proposed candidate substances in the extracts that could possibly mediate this effect. The study presented here could promote the use of this already known extract as a dietary supplement as a health promoting intervention in human nutrition.

## MATERIALS AND METHODS

### Algal extraction

*Eisenia bicyclis* extract was prepared according the method described by Onur et al. (Onur et al., 2013). In short, the dried algal material was grinded with an analytical mill (IKA Type A11 basic). 3 grams of grinded alga were transferred into a test tube containing 30 ml of boiling double distilled water. After slight stirring, the suspension was sonicated for 1 min and centrifuged for 2 min at 2000 g. The supernatant was filtered, and the soluble extract was freeze-dried and stored frozen until use.

### Fly husbandry

Wild type adult flies were kept as previously described [65]. In brief, they were cultivated on a diet containing 5 % (w/v) yeast extract, 8.6 % (w/v) corn meal, 5 % (w/v) sucrose, and 1 % (w/v) agar-agar supplemented with 1 % (v/v) propionic acid and 3 % (v/v) nipagin named here *Drosophila* medium. Adults, 3–5-days after hatching were used in the experiments. After this initial period of 3-5 days where the flies had the chance to mate, they were separated according to their sex. All experiments were performed at 25° C, a light-dark cycle of 12h:12h, and 60 % humidity. For most experiments, the wild type strain *w^1118^* was used. The following fly strains were used for the study: *w^1118^* (Bloomington stock ID #5905), *Sir2*-deficient (Bloomington stock ID #8838), *Tor*-deficient (Bloomington stock ID #11218), *dfoxo*-deficient (*foxo^21/21^*) (Marc Tatar lab), *y^1^w^1118^* (Bloomington stock ID #111281).

### Lifespan assay and analysis

The mated female flies were separated into experimental groups and kept on the food described above. Vials were regularly changed every 2 days and dead flies were counted every day. For the high sugar diet, the sucrose concentration was increased to 30 % and for the high-fat diet, the concentrated medium was supplemented with 20 % palm fat. To supplement the food with the algal extract, the aqueous extract was added on top of the food in the experimental vials at the final concentration of interest. For this reason, the concentration is calculated to correspond to the concentration in the entire medium. Then, the water is allowed to evaporate to dry the food. A total of at least 100 animals were used at minimum per experiment, divided into 5 aliquots with at least 20 animals each, which is well in the range used for lifespan assays [66].

### Body fat quantification

The whole-body triacylglycerol (TAG) content was measured using coupled colorimetric assay [67] as previously described [68]. In short, samples were collected (5 females per sample) and weighted. 1 ml PBS/Tween-20 (0.05 %) was added to the samples, and they were homogenized in a bead ruptor apparatus (BioLab Products, Bebensee, Germany) for 2 min at 3.25 m/s. Next, samples were centrifuged for 3 min at 3000 g and the supernatant was transferred to new tubes. The supernatant was heat-inactivated at 70° C for 10 min and centrifuged for 3 min at 2500 g. 50 μl of each sample was added to a 96-well plate and the absorbance was measured at 500 nm (T0). 200 μl prewarmed triglyceride reagent was added to each well and incubated for 30 min at 37° C with mild shaking. The absorbance was again measured at 500 nm (T1). The TAG concentration was determined by subtracting T0 from T1. The TAG content was quantified using a triolein-equivalent standard curve.

### Glucose measurement and protein content analysis

The body glucose levels were determined using a Glucose (HK) Assay Kit (GAHK-20, Sigma-Aldrich, Taufkirchen, Germany) according to the manufacturer’s instruction with minor modifications. Samples were collected (5 female flies per sample), weighted, and homogenized using a bead ruptor apparatus (BioLab Products, Bebensee, Germany) for 2 min at 3.25 m/s. For the glucose measurement, the supernatants were heated for 10 min at 70° C and then centrifuged for 3 min at maximum speed at 4° C. 30 μl of the supernatant was added to a well of a 96-well plate. 100 μl of the HK reagent was added to each well and the plate was incubated at room temperature for 15 min. Then, the absorbance was measured at 340 nm. The glucose content was calculated using a glucose standard curve. For the determination of the protein content, the samples were centrifuged for 1 min at 1000 rpm at 4C. The supernatant was transferred to a new 1.5 ml tube and centrifuged again at 6000 rpm, at 4° C for 10 min. The supernatant was again transferred to new tubes and centrifuged at maximum speed, at 4° C for 10 min. The protein content was measured using the Pierce BCA Protein Assay Kit according to the manufacturer’s instructions.

### Starvation, desiccation resistance, and fecundity assay

To assess flies’ resistance to starvation, flies transferred to vials containing 1 % agar after feeding with food of interest. The survivorship of flies was recorded every two h at 25° C and 60 % humidity and 12-h light-dark cycle. To determine the resistance to desiccation, flies were transferred to empty vials after feeding them with the food of interest. The flies were kept at 25° C and 60 % humidity and 12 h light-dark cycle. The survival of flies was monitored every 1 to 2 h. The mated female flies were kept in vials containing the food of interest and the laid eggs were counted every day for 7 days.

### Activity and sleep analysis

Activity and sleep were monitored using the *Drosophila* Activity Monitor (DAM2) system and analyzed by ShinyR [69]. The mated female flies were allowed to feed on the food of interest for 2 weeks and then were placed into tubes containing *Drosophila* medium. The tubes were loaded into the DAM system and maintained at constant conditions (25° C, 60 % humidity, and 12/12- h light-dark cycle). The first two days were spared for acclimatization and the data were collected on the third day. Sleep measurements were done according as described [69] with a period of 5 min without activity interpreted as a sleep phase. Data were recorded every 60 s.

### Food intake

The food consumption of flies was measured using the consumption-excretion method by Shell et al. [70]. First the 0.5 % (w/v) blue dyed (Brilliant Blue FCF food dye; E133) was prepared. The *Drosophila* medium or blue dyed *Drosophila* medium (0.5% (w/v) Brilliant Blue FCF food dye; E133) was dispensed into caps of 2 ml screw cap vials. For adaptation, individual flies were transferred to 2 ml screw cap vials with *Drosophila* medium. After a few h feeding on the concentrated medium the flies were transferred to 2 ml vials with blue dyed food. After 24 h feeding on blue dyed food three ceramic beads and 500 μl H_2_O were added to the vials. The samples were essentially treated and measured as described earlier [71].

### Puromycin assay and Western blotting

Young adult flies were starved overnight and transferred to a vial containing concentrated medium and puromycin (10 μg/ml). After 1 h treatment with the food, the flies were homogenized in 200 μl RIPA buffer using a bead ruptor apparatus (BioLab Products, Bebensee, Germany) for 2 min at 3.25 m/s. The protein concentration was quantified using BCA Protein Assay Kit (Thermofisher, Karlsruhe, Germany). 40 μg of protein was loaded on SDS-PAGE gel and the protein were resolved through electrophoresis. The proteins were transferred to a PVDF membrane using semi-dry transfer for 1 h. To block unspecific binding, the membrane was treated with 5 % non-fat dry milk powder in TBST for 1.30 h. The membrane was incubated with anti-puromycin (3RH11) antibody (Kerafast, Boston, USA, EQ0001) over night at 4° C. After 3 times washing with TBST, HRP-coupled anti-rabbit secondary antibody was used. The bands were visualized using Clarity™ Western ECL substrate and the ChemiDocImager (BioRad, Feldkirchen, Germany).

### Alpha-amylase activity

The alpha-amylase activity was determined using a high sensitivity alpha-amylase kit (K-AMYLSD, Megazyme Ltd., Bray, Ireland) according to the manufacturer’s instruction with minor modifications. 5 female flies per treatment group were homogenized in 200 μl PBS. 10 μl of the supernatants were added to 90 μl of amylase solution (65 μl amylase SD reagent mixed with 25 μl buffer). The assays were incubated for 30 min at 37° C, before adding 100 μl of stop reagent. For each sample, a blank (t = 0 min) was generated, where the stop solution was added prior to the amylase reagent. Absorbance of samples and corresponding blanks was read at 405 nm in a microplate reader. 4-5 samples per treatment group were measured in duplicate in two independent determinations. Amylase activities were calculated with the Mega-Calc™ Data Calculator sheet. One unit of amylase activity is defined as the amount of enzyme required to release one micromole of p-nitrophenol from 4,6-O-ethylidene-α-4-nitrophenyl-maltoheptaoside per min under the defined assay conditions.

### Untargeted data acquisition and HPLC-MS analyses

The untargeted data acquisition was performed using the TripleToF 6600 mass spectrometer (Sciex, Darmstadt, Germany) coupled to a Exion LC system (Sciex) operating in the ESI negative (ESI-) and ESI positive (ESI+) ionization mode as described earlier [72, 73]. Algae samples, QC and blank samples were analyzed using the data-independent acquisition (DIA) mode SWATH, by initially performing a ToF-MS scan with an accumulation time of 200 ms (m/z 100 - 1200), followed by 16 fixed Q1 isolation windows with an accumulation time of 81 ms in the m/z range from 100 to 1200 with 1 Da overlap. Following Q1 isolation windows were applied: m/z 169 – 240, 239 – 310, 309 – 380, 379 – 450, 449 – 520, 519 – 590, 589 – 660, 659 – 730, 729 – 800, 799 – 870, 869 – 940, 939 – 1010, 1009 – 1080, 1079 – 1150 and 1149 – 1200. The chromatographic separation was achieved on a C18 column with guard column of the same type (Kinetex C18, 100×2.1 mm, 1.7 μm, Phenomenex, Aschaffenburg, Germany) at an oven temperature of 40° C and a flow rate of 0.3 ml/min. The injection volume was 5 μl, the sample concentration was 10 mg extract per ml in water. Gradient elution with 0.1% formic acid (mobile phase A) and 0.1% formic acid in acetonitrile (mobile phase B) was as follows: 0 min 5% B, 0.5 min 5% B, 12 min 50% B, 13 min 100% B, 16.5 min 100% B, 17 min 5% B and 20 min 5% B. Samples were injected in triplicates in a randomized order with QC injections every eight to tenth injection. Blanks (solvent) were injected regularly, and the ToF calibration for instrument accuracy was performed after every fifth sample. For data acquisition and instrumental control, AnalystTF software (version 1.7.1, Sciex) was used.

Peak picking and feature alignment of acquired data in the ESI^-^ and ESI^+^ mode was performed using MS-DIAL (version 4.70) [74] (Supplementary Table 2). The feature alignment files for each ionization mode were then exported and further processed for feature reduction and annotation using MS-CleanR [75] (Supplementary Table 3) and MS-Finder (version 3.52) [76, 77] (Supplementary Table 4). For a first feature annotation process, public MS/MS libraries (Supplementary Table 4) were applied to obtain a broad overview of compounds. In a second annotation process, the seaweed specific Seaweed Metabolite Database (SWMD) was applied [78]. To make this library accessible for the MS-Finder annotation, the files storing the structural information (.mol) were downloaded from the SWMD homepage (https://www.swmd.co.in/) and converted to InChI Key, SMILES, formula, and exact mass using the Excel plugin from ChemOffice (version 20.0, PerkinElmer, Waltham, U.S.A). The structural information of the SWMD were further supplemented by compounds known to occur in brown algae (Supplementary Table 4).

After completion of the feature reduction and annotation process, an ESI^+^/ESI^-^ merged and annotated feature list was exported, which was further statistically analyzed using R. Therefore, NA values were first removed by inserting a half of the minimum observed intensity per feature for all features, then the dataset was log10 transformed and pareto-scaled. For a first overview a principal component analysis (PCA) was performed using the R package mixomics [79] (Figure 6A). To gather further insight, which specific features are responsible for the separation of the algae samples, a partial least-squares discriminant analysis (PLS-DA) was performed using the mixomics package (Figure 6B). A special focus was set on features higher abundant in the *Eisenia* samples compared to the *Salicornia* and *Saccorhiza* samples. Therefore, *Salicornia* and *Saccorhiza* samples were put together in one group and compared to *Eisenia* samples. The PLS-DA was then calculated using two components, while the background was calculated using the maximum distance function and a resolution of 500. The loadings were subsequently exported and sorted by increasing values of component 1 to select only the first 100 features, which are higher concentrated in *Eisenia* compared to *Salicornia/Saccorhiza* samples. Based on the two annotation processes using MS-CleanR/MS-Finder, features are shown with up to two annotations (Supplementary Table 5).

### Statistical analysis

Statistical analyses were performed with the Prism 9 work package. For analyses of lifespan data, a log-rank test was used (Mantel-Cox or Gehan-Breslow-Wilcoxon). At least 100 individuals were analyzed per treatment, usually in 5 replicates with at least 20 individuals each. Parametric data were analyzed with unpaired Student’s *t*-test and non-parametric data with Mann–Whitney test (usually with N≥10).

## Supplementary Material

Supplementary Figures

Supplementary Tables 1-4

Supplementary Table 5

## References

[1] Kapahi P, Kaeberlein M, Hansen M. Dietary restriction and lifespan: Lessons from invertebrate models. Ageing Res Rev. 2017; 39:3–14. 10.1016/j.arr.2016.12.005 28007498 PMC5476520

[2] Mattison JA, Colman RJ, Beasley TM, Allison DB, Kemnitz JW, Roth GS, Ingram DK, Weindruch R, de Cabo R, Anderson RM. Caloric restriction improves health and survival of rhesus monkeys. Nat Commun. 2017; 8:14063. 10.1038/ncomms14063 28094793 PMC5247583

[3] Ingram DK, Zhu M, Mamczarz J, Zou S, Lane MA, Roth GS, deCabo R. Calorie restriction mimetics: an emerging research field. Aging Cell. 2006; 5:97–108. 10.1111/j.1474-9726.2006.00202.x 16626389

[4] Vézina C, Kudelski A, Sehgal SN. Rapamycin (AY-22,989), a new antifungal antibiotic. I. Taxonomy of the producing streptomycete and isolation of the active principle. J Antibiot (Tokyo). 1975; 28:721–6. 10.7164/antibiotics.28.721 1102508

[5] Bjedov I, Toivonen JM, Kerr F, Slack C, Jacobson J, Foley A, Partridge L. Mechanisms of life span extension by rapamycin in the fruit fly Drosophila melanogaster. Cell Metab. 2010; 11:35–46. 10.1016/j.cmet.2009.11.010 20074526 PMC2824086

[6] Harrison DE, Strong R, Sharp ZD, Nelson JF, Astle CM, Flurkey K, Nadon NL, Wilkinson JE, Frenkel K, Carter CS, Pahor M, Javors MA, Fernandez E, Miller RA. Rapamycin fed late in life extends lifespan in genetically heterogeneous mice. Nature. 2009; 460:392–5. 10.1038/nature08221 19587680 PMC2786175

[7] Bitto A, Ito TK, Pineda VV, LeTexier NJ, Huang HZ, Sutlief E, Tung H, Vizzini N, Chen B, Smith K, Meza D, Yajima M, Beyer RP, et al. Transient rapamycin treatment can increase lifespan and healthspan in middle-aged mice. Elife. 2016; 5:e16351. 10.7554/eLife.16351 27549339 PMC4996648

[8] Blagosklonny MV. Rapamycin for longevity: opinion article. Aging (Albany NY). 2019; 11:8048–67. 10.18632/aging.102355 31586989 PMC6814615

[9] Rhoads TW, Anderson RM. Alpha-Ketoglutarate, the Metabolite that Regulates Aging in Mice. Cell Metab. 2020; 32:323–5. 10.1016/j.cmet.2020.08.009 32877686 PMC8191137

[10] Asadi Shahmirzadi A, Edgar D, Liao CY, Hsu YM, Lucanic M, Asadi Shahmirzadi A, Wiley CD, Gan G, Kim DE, Kasler HG, Kuehnemann C, Kaplowitz B, Bhaumik D, et al. Alpha-Ketoglutarate, an Endogenous Metabolite, Extends Lifespan and Compresses Morbidity in Aging Mice. Cell Metab. 2020; 32:447–56.e6. 10.1016/j.cmet.2020.08.004 32877690 PMC8508957

[11] Chin RM, Fu X, Pai MY, Vergnes L, Hwang H, Deng G, Diep S, Lomenick B, Meli VS, Monsalve GC, Hu E, Whelan SA, Wang JX, et al. The metabolite α-ketoglutarate extends lifespan by inhibiting ATP synthase and TOR. Nature. 2014; 510:397–401. 10.1038/nature13264 24828042 PMC4263271

[12] Su Y, Wang T, Wu N, Li D, Fan X, Xu Z, Mishra SK, Yang M. Alpha-ketoglutarate extends Drosophila lifespan by inhibiting mTOR and activating AMPK. Aging (Albany NY). 2019; 11:4183–97. 10.18632/aging.102045 31242135 PMC6629006

[13] Johnson SC, Rabinovitch PS, Kaeberlein M. mTOR is a key modulator of ageing and age-related disease. Nature. 2013; 493:338–45. 10.1038/nature11861 23325216 PMC3687363

[14] Murray M, Dordevic AL, Ryan L, Bonham MP. An emerging trend in functional foods for the prevention of cardiovascular disease and diabetes: Marine algal polyphenols. Crit Rev Food Sci Nutr. 2018; 58:1342–58. 10.1080/10408398.2016.1259209 27834493

[15] Willcox DC, Scapagnini G, Willcox BJ. Healthy aging diets other than the Mediterranean: a focus on the Okinawan diet. Mech Ageing Dev. 2014; 136–137:148–62. 10.1016/j.mad.2014.01.002 24462788 PMC5403516

[16] Nguyen TT, Caito SW, Zackert WE, West JD, Zhu S, Aschner M, Fessel JP, Roberts LJ 2nd. Scavengers of reactive γ-ketoaldehydes extend Caenorhabditis elegans lifespan and healthspan through protein-level interactions with SIR-2.1 and ETS-7. Aging (Albany NY). 2016; 8:1759–80. 10.18632/aging.101011 27514077 PMC5032694

[17] Gardner TS. The use of Drosophila melanogaster as a screening agent for longevity factors; pantothenic acid as a longevity factor in royal jelly. J Gerontol. 1948; 3:1–8. 10.1093/geronj/3.1.1 18856647

[18] Ye X, Linton JM, Schork NJ, Buck LB, Petrascheck M. A pharmacological network for lifespan extension in Caenorhabditis elegans. Aging Cell. 2014; 13:206–15. 10.1111/acel.12163 24134630 PMC3955372

[19] Grünwald S, Stellzig J, Adam IV, Weber K, Binger S, Boll M, Knorr E, Twyman RM, Vilcinskas A, Wenzel U. Longevity in the red flour beetle Tribolium castaneum is enhanced by broccoli and depends on nrf-2, jnk-1 and foxo-1 homologous genes. Genes Nutr. 2013; 8:439–48. 10.1007/s12263-012-0330-6 23321956 PMC3755130

[20] Chien S, Reiter LT, Bier E, Gribskov M. Homophila: human disease gene cognates in Drosophila. Nucleic Acids Res. 2002; 30:149–51. 10.1093/nar/30.1.149 11752278 PMC99119

[21] Hoffmann J, Romey R, Fink C, Roeder T. Drosophila as a model to study metabolic disorders. Adv Biochem Eng Biotechnol. 2013; 135:41–61. 10.1007/10_2013_196 23604212

[22] Li Y, Romey-Glüsing R, Tahan Zadeh N, von Frieling J, Hoffmann J, Huebbe P, Bruchhaus I, Rimbach G, Fink C, Roeder T. Furbellow (Brown Algae) Extract Increases Lifespan in Drosophila by Interfering with TOR-Signaling. Nutrients. 2020; 12:1172. 10.3390/nu12041172 32331413 PMC7230866

[23] Huebbe P, Nikolai S, Schloesser A, Herebian D, Campbell G, Glüer CC, Zeyner A, Demetrowitsch T, Schwarz K, Metges CC, Roeder T, Schultheiss G, Ipharraguerre IR, Rimbach G. An extract from the Atlantic brown algae Saccorhiza polyschides counteracts diet-induced obesity in mice via a gut related multi-factorial mechanisms. Oncotarget. 2017; 8:73501–15. 10.18632/oncotarget.18113 29088722 PMC5650277

[24] Whitaker DM, Carlson GP. Anti-inflammation mechanism of extract from Eisenia bicyclis (Kjellman) Setchell. J Pharm Sci. 1975; 64:1258–9. 10.1002/jps.2600640735 1151693

[25] Kim KA, Kim SM, Kang SW, Jeon SI, Um BH, Jung SH. Edible seaweed, Eisenia bicyclis, protects retinal ganglion cells death caused by oxidative stress. Mar Biotechnol (NY). 2012; 14:383–95. 10.1007/s10126-012-9459-y 22610700

[26] Onur S, Stöckmann H, Zenthoefer M, Piker L, Döring F. The Plant Extract Collection Kiel in Schleswig-Holstein (PECKISH) Is an Open Access Screening Library. J Food Res. 2013; 2:101–6. 10.5539/jfr.v2n4p101

[27] Thompson JB, Su OO, Yang N, Bauer JH. Sleep-length differences are associated with altered longevity in the fruit fly Drosophila melanogaster. Biol Open. 2020; 9:bio054361. 10.1242/bio.054361 32938639 PMC7520458

[28] Eom SH, Lee SH, Yoon NY, Jung WK, Jeon YJ, Kim SK, Lee MS, Kim YM. α-Glucosidase- and α-amylase-inhibitory activities of phlorotannins from Eisenia bicyclis. J Sci Food Agric. 2012; 92:2084–90. 10.1002/jsfa.5585 22271637

[29] Hoffmann J, Romey R, Fink C, Yong L, Roeder T. Overexpression of Sir2 in the adult fat body is sufficient to extend lifespan of male and female Drosophila. Aging (Albany NY). 2013; 5:315–27. 10.18632/aging.100553 23765091 PMC3651523

[30] Robida-Stubbs S, Glover-Cutter K, Lamming DW, Mizunuma M, Narasimhan SD, Neumann-Haefelin E, Sabatini DM, Blackwell TK. TOR signaling and rapamycin influence longevity by regulating SKN-1/Nrf and DAF-16/FoxO. Cell Metab. 2012; 15:713–24. 10.1016/j.cmet.2012.04.007 22560223 PMC3348514

[31] Liu JJ, Zhao GX, He LL, Wang Z, Zibrila AI, Niu BC, Gong HY, Xu JN, Soong L, Li CF, Lu Y. Lycium barbarum polysaccharides inhibit ischemia/reperfusion-induced myocardial injury via the Nrf2 antioxidant pathway. Toxicol Rep. 2021; 8:657–67. 10.1016/j.toxrep.2021.03.019 33868952 PMC8041662

[32] Long J, Gao H, Sun L, Liu J, Zhao-Wilson X. Grape extract protects mitochondria from oxidative damage and improves locomotor dysfunction and extends lifespan in a Drosophila Parkinson’s disease model. Rejuvenation Res. 2009; 12:321–31. 10.1089/rej.2009.0877 19929256

[33] Peng C, Zuo Y, Kwan KM, Liang Y, Ma KY, Chan HY, Huang Y, Yu H, Chen ZY. Blueberry extract prolongs lifespan of Drosophila melanogaster. Exp Gerontol. 2012; 47:170–8. 10.1016/j.exger.2011.12.001 22197903

[34] Strilbytska OM, Zayachkivska A, Koliada A, Galeotti F, Volpi N, Storey KB, Vaiserman A, Lushchak O. Anise Hyssop Agastache foeniculum Increases Lifespan, Stress Resistance, and Metabolism by Affecting Free Radical Processes in Drosophila. Front Physiol. 2020; 11:596729. 10.3389/fphys.2020.596729 33391017 PMC7772399

[35] Grandison RC, Wong R, Bass TM, Partridge L, Piper MD. Effect of a standardised dietary restriction protocol on multiple laboratory strains of Drosophila melanogaster. PLoS One. 2009; 4:e4067. 10.1371/journal.pone.0004067 19119322 PMC2607010

[36] Sampathkumar NK, Bravo JI, Chen Y, Danthi PS, Donahue EK, Lai RW, Lu R, Randall LT, Vinson N, Benayoun BA. Widespread sex dimorphism in aging and age-related diseases. Hum Genet. 2020; 139:333–56. 10.1007/s00439-019-02082-w 31677133 PMC7031050

[37] Hudry B, de Goeij E, Mineo A, Gaspar P, Hadjieconomou D, Studd C, Mokochinski JB, Kramer HB, Plaçais PY, Preat T, Miguel-Aliaga I. Sex Differences in Intestinal Carbohydrate Metabolism Promote Food Intake and Sperm Maturation. Cell. 2019; 178:901–18.e16. 10.1016/j.cell.2019.07.029 31398343 PMC6700282

[38] Wat LW, Chao C, Bartlett R, Buchanan JL, Millington JW, Chih HJ, Chowdhury ZS, Biswas P, Huang V, Shin LJ, Wang LC, Gauthier ML, Barone MC, et al. A role for triglyceride lipase brummer in the regulation of sex differences in Drosophila fat storage and breakdown. PLoS Biol. 2020; 18:e3000595. 10.1371/journal.pbio.3000595 31961851 PMC6994176

[39] Magwere T, Chapman T, Partridge L. Sex differences in the effect of dietary restriction on life span and mortality rates in female and male Drosophila melanogaster. J Gerontol A Biol Sci Med Sci. 2004; 59:3–9. 10.1093/gerona/59.1.b3 14718480

[40] Bonawitz ND, Chatenay-Lapointe M, Pan Y, Shadel GS. Reduced TOR signaling extends chronological life span via increased respiration and upregulation of mitochondrial gene expression. Cell Metab. 2007; 5:265–77. 10.1016/j.cmet.2007.02.009 17403371 PMC3460550

[41] Kapahi P, Chen D, Rogers AN, Katewa SD, Li PW, Thomas EL, Kockel L. With TOR, less is more: a key role for the conserved nutrient-sensing TOR pathway in aging. Cell Metab. 2010; 11:453–65. 10.1016/j.cmet.2010.05.001 20519118 PMC2885591

[42] Kapahi P, Zid BM, Harper T, Koslover D, Sapin V, Benzer S. Regulation of lifespan in Drosophila by modulation of genes in the TOR signaling pathway. Curr Biol. 2004; 14:885–90. 10.1016/j.cub.2004.03.059 15186745 PMC2754830

[43] Martins R, Lithgow GJ, Link W. Long live FOXO: unraveling the role of FOXO proteins in aging and longevity. Aging Cell. 2016; 15:196–207. 10.1111/acel.12427 26643314 PMC4783344

[44] Antikainen H, Driscoll M, Haspel G, Dobrowolski R. TOR-mediated regulation of metabolism in aging. Aging Cell. 2017; 16:1219–33. 10.1111/acel.12689 28971552 PMC5676073

[45] Mazzotti DR, Guindalini C, Moraes WA, Andersen ML, Cendoroglo MS, Ramos LR, Tufik S. Human longevity is associated with regular sleep patterns, maintenance of slow wave sleep, and favorable lipid profile. Front Aging Neurosci. 2014; 6:134. 10.3389/fnagi.2014.00134 25009494 PMC4067693

[46] McKillop LE, Vyazovskiy VV. Sleep and ageing: from human studies to rodent models. Curr Opin Physiol. 2020; 15:210–6. 10.1016/j.cophys.2020.03.004 32467862 PMC7255885

[47] Vaccaro A, Kaplan Dor Y, Nambara K, Pollina EA, Lin C, Greenberg ME, Rogulja D. Sleep Loss Can Cause Death through Accumulation of Reactive Oxygen Species in the Gut. Cell. 2020; 181:1307–28.e15. 10.1016/j.cell.2020.04.049 32502393

[48] Jung HA, Jin SE, Ahn BR, Lee CM, Choi JS. Anti-inflammatory activity of edible brown alga Eisenia bicyclis and its constituents fucosterol and phlorotannins in LPS-stimulated RAW264.7 macrophages. Food Chem Toxicol. 2013; 59:199–206. 10.1016/j.fct.2013.05.061 23774261

[49] Kang YM, Eom SH, Kim YM. Protective effect of phlorotannins from Eisenia bicyclis against lipopolysaccharide-stimulated inflammation in HepG2 cells. Environ Toxicol Pharmacol. 2013; 35:395–401. 10.1016/j.etap.2013.01.009 23454824

[50] Irfan M, Kwon TH, Yun BS, Park NH, Rhee MH. Eisenia bicyclis (brown alga) modulates platelet function and inhibits thrombus formation via impaired P2Y12 receptor signaling pathway. Phytomedicine. 2018; 40:79–87. 10.1016/j.phymed.2018.01.003 29496178

[51] Menshova RV, Ermakova SP, Anastyuk SD, Isakov VV, Dubrovskaya YV, Kusaykin MI, Um BH, Zvyagintseva TN. Structure, enzymatic transformation and anticancer activity of branched high molecular weight laminaran from brown alga Eisenia bicyclis. Carbohydr Polym. 2014; 99:101–9. 10.1016/j.carbpol.2013.08.037 24274485

[52] Kwon TH, Kim TW, Kim CG, Park NH. Antioxidant activity of various solvent fractions from edible brown alga, Eisenia bicyclis and its active compounds. J Food Sci. 2013; 78:C679–84. 10.1111/1750-3841.12109 23557350

[53] Choi JS, Han YR, Byeon JS, Choung SY, Sohn HS, Jung HA. Protective effect of fucosterol isolated from the edible brown algae, Ecklonia stolonifera and Eisenia bicyclis, on tert-butyl hydroperoxide- and tacrine-induced HepG2 cell injury. J Pharm Pharmacol. 2015; 67:1170–8. 10.1111/jphp.12404 25773602

[54] Eom SH, Lee MS, Lee EW, Kim YM, Kim TH. Pancreatic lipase inhibitory activity of phlorotannins isolated from Eisenia bicyclis. Phytother Res. 2013; 27:148–51. 10.1002/ptr.4694 22473750

[55] Kim HJ, Dasagrandhi C, Kim SH, Kim BG, Eom SH, Kim YM. *In Vitro* Antibacterial Activity of Phlorotannins from Edible Brown Algae, Eisenia bicyclis Against Streptomycin-Resistant Listeria monocytogenes. Indian J Microbiol. 2018; 58:105–8. 10.1007/s12088-017-0693-x 29434404 PMC5801182

[56] Kim SM, Kang K, Jeon JS, Jho EH, Kim CY, Nho CW, Um BH. Isolation of phlorotannins from Eisenia bicyclis and their hepatoprotective effect against oxidative stress induced by tert-butyl hyperoxide. Appl Biochem Biotechnol. 2011; 165:1296–307. 10.1007/s12010-011-9347-3 21892616

[57] Eom SH, Kim DH, Lee SH, Yoon NY, Kim JH, Kim TH, Chung YH, Kim SB, Kim YM, Kim HW, Lee MS, Kim YM. *In vitro* antibacterial activity and synergistic antibiotic effects of phlorotannins isolated from Eisenia bicyclis against methicillin-resistant Staphylococcus aureus. Phytother Res. 2013; 27:1260–4. 10.1002/ptr.4851 23042620

[58] Jung HA, Roy A, Jung JH, Choi JS. Evaluation of the inhibitory effects of eckol and dieckol isolated from edible brown alga Eisenia bicyclis on human monoamine oxidases A and B. Arch Pharm Res. 2017; 40:480–91. 10.1007/s12272-017-0904-3 28251489

[59] Gunathilaka TL, Samarakoon K, Ranasinghe P, Peiris LDC. Antidiabetic Potential of Marine Brown Algae-a Mini Review. J Diabetes Res. 2020; 2020:1230218. 10.1155/2020/1230218 32377517 PMC7197011

[60] Lee SH, Jeon YJ. Anti-diabetic effects of brown algae derived phlorotannins, marine polyphenols through diverse mechanisms. Fitoterapia. 2013; 86:129–36. 10.1016/j.fitote.2013.02.013 23466874

[61] Jung HA, Islam MN, Lee CM, Oh SH, Lee S, Jung JH, Choi JS. Kinetics and molecular docking studies of an anti-diabetic complication inhibitor fucosterol from edible brown algae Eisenia bicyclis and Ecklonia stolonifera. Chem Biol Interact. 2013; 206:55–62. 10.1016/j.cbi.2013.08.013 23994501

[62] John M, Gumbinger HG, Winterhoff H. Oxidation products of caffeic acid as model substances for the antigonadotropic activity of plant extracts. Planta Med. 1990; 56:14–8. 10.1055/s-2006-960874 2356237

[63] Li JQ, Fang JS, Qin XM, Gao L. Metabolomics profiling reveals the mechanism of caffeic acid in extending lifespan in Drosophila melanogaster. Food Funct. 2020; 11:8202–13. 10.1039/d0fo01332c 32966485

[64] Ku CS, Pham TX, Park Y, Kim B, Shin MS, Kang I, Lee J. Edible blue-green algae reduce the production of pro-inflammatory cytokines by inhibiting NF-κB pathway in macrophages and splenocytes. Biochim Biophys Acta. 2013; 1830:2981–8. 10.1016/j.bbagen.2013.01.018 23357040 PMC3594481

[65] Romey-Glüsing R, Li Y, Hoffmann J, von Frieling J, Knop M, Pfefferkorn R, Bruchhaus I, Fink C, Roeder T. Nutritional regimens with periodically recurring phases of dietary restriction extend lifespan in Drosophila. FASEB J. 2018; 32:1993–2003. 10.1096/fj.201700934R 29196499

[66] Piper MD, Partridge L. Protocols to Study Aging in Drosophila. Methods Mol Biol. 2016; 1478:291–302. 10.1007/978-1-4939-6371-3_18 27730590 PMC5507281

[67] Hildebrandt A, Bickmeyer I, Kühnlein RP. Reliable Drosophila body fat quantification by a coupled colorimetric assay. PLoS One. 2011; 6:e23796. 10.1371/journal.pone.0023796 21931614 PMC3170289

[68] Proske A, Bossen J, von Frieling J, Roeder T. Low-protein diet applied as part of combination therapy or stand-alone normalizes lifespan and tumor proliferation in a model of intestinal cancer. Aging (Albany NY). 2021; 13:24017–36. 10.18632/aging.203692 34766923 PMC8610115

[69] Cichewicz K, Hirsh J. ShinyR-DAM: a program analyzing Drosophila activity, sleep and circadian rhythms. Commun Biol. 2018; 1:25. 10.1038/s42003-018-0031-9 29911688 PMC6002956

[70] Shell BC, Schmitt RE, Lee KM, Johnson JC, Chung BY, Pletcher SD, Grotewiel M. Measurement of solid food intake in Drosophila via consumption-excretion of a dye tracer. Sci Rep. 2018; 8:11536. 10.1038/s41598-018-29813-9 30068981 PMC6070562

[71] Finetti L, Tiedemann L, Zhang X, Civolani S, Bernacchia G, Roeder T. Monoterpenes alter TAR1-driven physiology in Drosophila species. J Exp Biol. 2021; 224:jeb232116. 10.1242/jeb.232116 33234680

[72] Dirndorfer S, Hammerl R, Kitajima S, Kitada R, Frank O, Dunkel A, Hofmann T. Identification and Quantitation of Taste-Active Compounds in Dried Scallops by Combined Application of the Sensomics and a Quantitative NMR Approach. J Agric Food Chem. 2022; 70:247–59. 10.1021/acs.jafc.1c05257 34965128

[73] Sebald K, Dunkel A, Hofmann T. Mapping Taste-Relevant Food Peptidomes by Means of Sequential Window Acquisition of All Theoretical Fragment Ion-Mass Spectrometry. J Agric Food Chem. 2020; 68:10287–98. 10.1021/acs.jafc.9b04581 31508943

[74] Tsugawa H, Cajka T, Kind T, Ma Y, Higgins B, Ikeda K, Kanazawa M, VanderGheynst J, Fiehn O, Arita M. MS-DIAL: data-independent MS/MS deconvolution for comprehensive metabolome analysis. Nat Methods. 2015; 12:523–6. 10.1038/nmeth.3393 25938372 PMC4449330

[75] Fraisier-Vannier O, Chervin J, Cabanac G, Puech V, Fournier S, Durand V, Amiel A, André O, Benamar OA, Dumas B, Tsugawa H, Marti G. MS-CleanR: A Feature-Filtering Workflow for Untargeted LC-MS Based Metabolomics. Anal Chem. 2020; 92:9971–81. 10.1021/acs.analchem.0c01594 32589017

[76] Tsugawa H, Kind T, Nakabayashi R, Yukihira D, Tanaka W, Cajka T, Saito K, Fiehn O, Arita M. Hydrogen Rearrangement Rules: Computational MS/MS Fragmentation and Structure Elucidation Using MS-FINDER Software. Anal Chem. 2016; 88:7946–58. 10.1021/acs.analchem.6b00770 27419259 PMC7063832

[77] Lai Z, Tsugawa H, Wohlgemuth G, Mehta S, Mueller M, Zheng Y, Ogiwara A, Meissen J, Showalter M, Takeuchi K, Kind T, Beal P, Arita M, Fiehn O. Identifying metabolites by integrating metabolome databases with mass spectrometry cheminformatics. Nat Methods. 2018; 15:53–6. 10.1038/nmeth.4512 29176591 PMC6358022

[78] Davis GD, Vasanthi AH. Seaweed metabolite database (SWMD): A database of natural compounds from marine algae. Bioinformation. 2011; 5:361–4. 10.6026/97320630005361 21423723 PMC3053594

[79] Rohart F, Gautier B, Singh A, Lê Cao KA. mixOmics: An R package for ‘omics feature selection and multiple data integration. PLoS Comput Biol. 2017; 13:e1005752. 10.1371/journal.pcbi.1005752 29099853 PMC5687754

