# Supplementary material for: An aqueous extract of the brown alga Eisenia bicyclis extends lifespan in a sex-specific manner by interfering with the Tor-FoxO axis
Source: Aging (Albany NY). 2022 Aug 16;14(16):6427–48. doi: 10.18632/aging.204218 (PMC9467403; doi:10.18632/aging.204218)
Supplement: Supplementary Figures [file aging-14-204218-s001.pdf]

SUPPLEMENTARY FIGURES

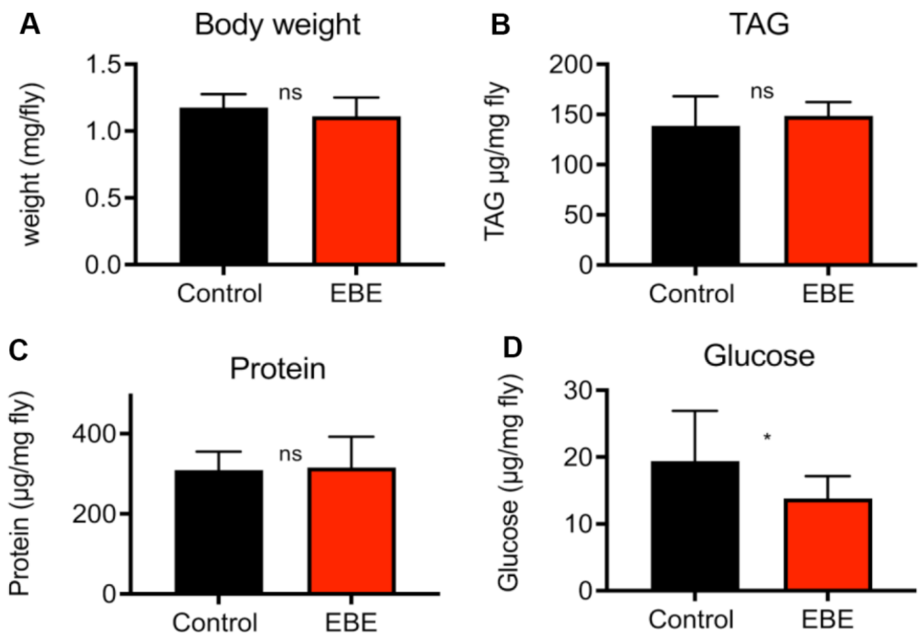

Supplementary Figure 1. Effect of *Eisenia bicyclis* extracts on female flies subjected to a HSD. Body weight (**A**), TAG levels (**B**), protein contents (**C**), and glucose contents of females on high sugar control diet and high sugar diets supplemented with 0.05 %. N≥10, mean values ± S.E.M. are given (**D**). Statistical analyses were performed with unpaired t-tests. ns means not significant, * means p< 0.05.

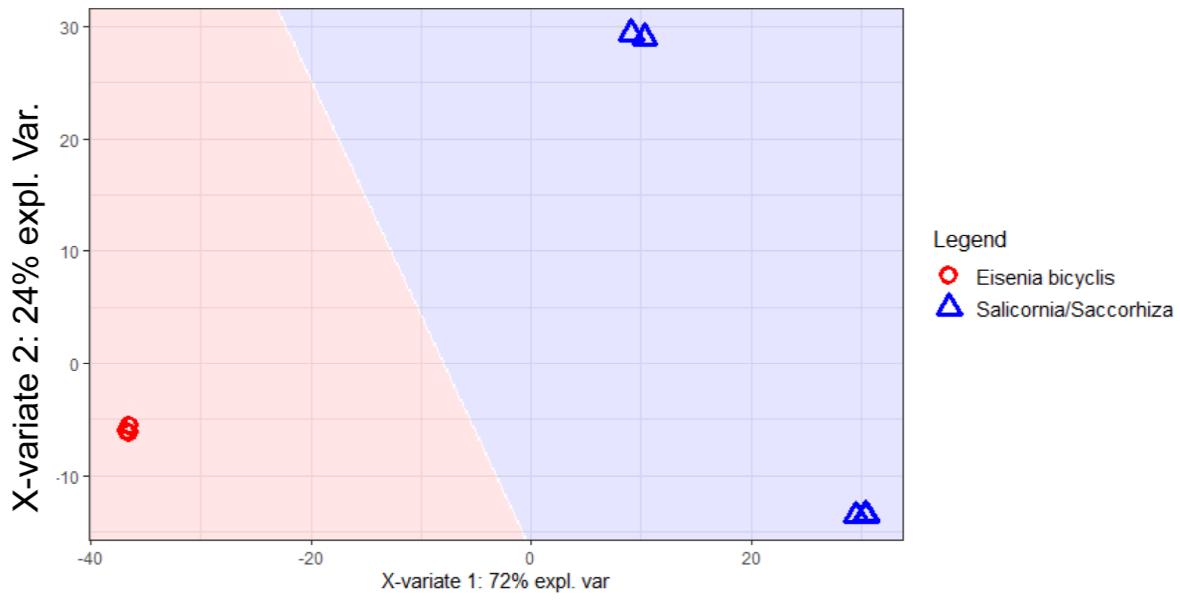

Supplementary Figure 2. Untargeted metabolome analysis of algae extracts. Partial least-squares discriminant analysis of algae extracts measured using the ESI+ and ESI- acquisition mode. Underlying peak areas are log10 transformed and pareto-scaled. The Top100 features responsible for the separation of *Eisenia bicyclis* from *Salicornia/Saccorhiza* were extracted for further analysis (Supplementary Table 4).

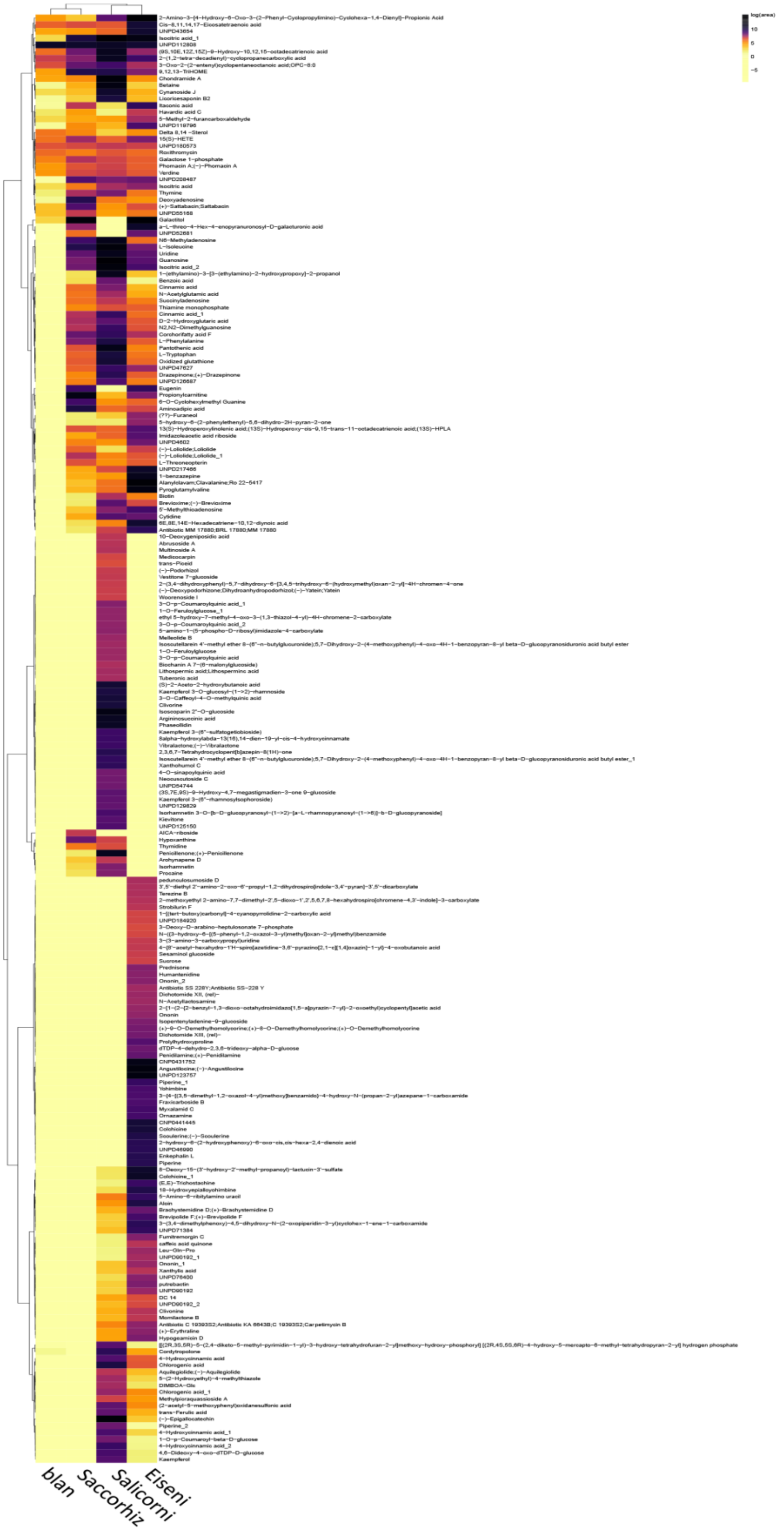

Supplementary Figure 3. Heatmap of metabolome analysis of algae extracts. Relative abundances of the Top100 compounds showing differential distribution between the samples (*Eisenia bicyclis*-, *Salicornia spec.*-, *Saccorhiza polyschides*-extract).
