# Supplementary material for: An aqueous extract of the brown alga Eisenia bicyclis extends lifespan in a sex-specific manner by interfering with the Tor-FoxO axis
Source: Aging (Albany NY). 2022 Aug 16;14(16):6427–48. doi: 10.18632/aging.204218 (PMC9467403; doi:10.18632/aging.204218)
Supplement: Supplementary Tables 1-4 [file aging-14-204218-s002.pdf]

SUPPLEMENTARY TABLES

Supplementary Table 1. Median, mean, and maximum lifespans of experiments described in this manuscript.

		**Median lifespan[d]**	**Mean lifespan [d]**	**Maximum lifespan [d]**
Drosophila medium	EBE 0.05 %	56	48.75	66
	control	40	39.46	59
	EBE 0.1 %	53	52.5	65
	control	38	38.18	53
Male flies	EBE 0.05 %	51	49.15	65
	Control	53.5	50.33	65
yw	EBE 0.05 %	39	36.65	52
	control	33	32.17	45
Starvation	EBE 0.05 %	72 h	70.29 h	96 h
	control	70 h	68.62 h	94 h
Desiccation	EBE 0.05 %	25 h	26.49 h	38 h
	control	24 h	24.39 h	38 h
HFD	EBE 0.05 %	30	26.7	39
	control	29	25.39	36
HSD	EBE 0.05 %	46	42.12	54
	control	35	34.3	50
dSir2-deficient	EBE 0.05 %	41	36.61	51
	control	37	34.12	44
Tor-deficient	EBE 0.05 %	48	47.81	60
	control	48	46.86	58
Foxo-deficient	EBE 0.05 %	26	26.82	48
	control	26	24.83	43
		Median lifespan [h]	Mean lifespan [h]	maximum lifespan [h]
Starvation	EBE 0.05 %	72	70.29	96
	control	70	68.62	94
Desiccation	EBE 0.05 %	25	26.49	38
	control	24	24.39	38

Supplementary Table 2. Data processing parameters using MS-Dial 4.70.

**Data collection**	
Retention time begin	0 min
Retention time end	20 min
MS1 mass range begin	100 Da
MS1 mass range end	1200 Da
MS/MS mass range begin	50 Da
MS/MS mass range end	1200 Da
**Peak detection**	
Minimum peak height	1000 amplitude
**Adduct (neg)**	
[M-H]-, [M-H-H2O]-, [M+Na-2H]-, [M+FA-H]-, [2M-H]-, [2M+FA-H]-	
**Alignment**	
Retention time tolerance	0.1 min
MS1 tolerance	0.025 Da
Remove features based on blank information	yes

Preset values were applied if not stated otherwise.

Supplementary Table 3. Data processing parameters using MS-CleanR 1.0 with R 3.6.1 (x64).

**Clean MS-Dial data**	
Maximum mass difference	0.005 Da
Maximum retention time difference	0.01 min
Use Pearson correlation to compute clusters	no
**Keep top peaks by cluster**	
Intensity	yes
Degree	yes
Number of peaks to keep	2
**Launch MS-Finder annotation**	
Select the best annotation for each peak based only on MS-Finder scores?	yes

Preset values were applied if not stated otherwise.

Supplementary Table 4. Data processing parameters using MS-Finder 3.52.

**Method**	
Spectral database search	yes
Formula prediction and structure elucidation by *in silico* fragmenter	yes
Use internal experimental library (MassBank, GNPS, ReSpect	yes
User-defined DB	All public MS/MS database 13.04.2021*
Precursor oriented spectral search	yes
**Basic**	
Mass tolerance (MS1)	0.01 Da
Mass tolerance (MS2)	0.05 Da
Mass range max	1200 Da
Mass range min	50 Da
**Formula Finder**	
Element selection	O, N, P, S, F, Cl, Br, I
Time out	1 min
**Data source**	
Local Databases	LipidMAPS, YMDB, ECMDB, DrugBank, FooDB, PlantCyc, CheBI, T3DB, STOFF, NPA, NANPDB, COCONUT, KNApSAcK, PubChem, UNPD
User-defined DB	InChI and SMILES information of SWMD compounds supplemented by Eisenin, Pyropheophytin a, Triphlorethol A, 7-Phloroeckol, Eiseniachloride-A-C, Eiseniaiodide-A-B
MINEs, PubChem Online	Never use it

*http://prime.psc.riken.jp/compms/msdial/main.html#MSP
Preset values were applied if not stated otherwise.
