# Supplementary material for: An aqueous extract of the brown alga Eisenia bicyclis extends lifespan in a sex-specific manner by interfering with the Tor-FoxO axis
Source: Aging (Albany NY). 2022 Aug 16;14(16):6427–48. doi: 10.18632/aging.204218 (PMC9467403; doi:10.18632/aging.204218)
Supplement: Supplementary Table 5 [file aging-14-204218-s003.docx]

**Supplementary Table 5. Top100 features responsible for the separation of Eisenia from Salicornia/Saccorhiza.**

| *Top100* | *Feature ID* | *Rt [min]* | *m/z* | *Annotated Adduct* | *Annotated Structure** | *Top100* | *Feature ID* | *Rt [min]* | *m/z* | *Annotated Adduct* | *Annotated Structure** |
| --- | --- | --- | --- | --- | --- | --- | --- | --- | --- | --- | --- |
| *1* | *pos_1670* | *5.193* | *277.11807* | *[M+H]+* | *5-Amino-6-ribitylamino uracil* | *52* | *neg_3155* | *2.014* | *583.03546* | *[M-H]-* |  |
| *2* | *pos_1954* | *2.96* | *302.20639* | *[M+H]+* | *DC 14* | *53* | *neg_1833* | *7.378* | *369.0257* | *[M-H]-* | *UNPD193816* |
| *3* | *neg_564* | *1.804* | *213.08812* | *[M-H]-* | *Ichthyotherol* | *54* | *neg_1592* | *1.654* | *341.08591* | *[M-H]-* | *1-O-Caffeoylglucose* |
| *4* | *pos_1614* | *1.333* | *272.12149* | *[M+H]+* |  | *55* | *neg_518* | *1* | *207.0265* | *[M-H]-* | *2-(2'-Methylthio)ethylmalic acid* |
| *5* | *neg_1838* | *0.978* | *370.05988* | *[M-H]-* | *N-(4-ACETYLPHENYL)-5-(5-CHLORO-2,4-DIHYDROXYPHENYL)-1H-PYRAZOLE-4-CARBOXAMIDE* | *56* | *neg_2854* | *5.186* | *513.02795* | *[M-H]-* |  |
| *6* | *pos_1303* | *1.299* | *242.09982* | *[M+H]+* | *UNPD71384* | *57* | *pos_3389* | *7.135* | *743.09229* | *[M+H]+* |  |
| *7* | *pos_1322* | *4.256* | *243.13374* | *[M+H]+* | *UNPD228404* | *58* | *pos_1337* | *0.984* | *244.54636* | *[M+2H]2+* | *2-pyridin-4-yl-N-[4-(2-thiazolylsulfamoyl)phenyl]-4-quinolinecarboxamide* |
| *8* | *pos_615* | *3.691* | *178.1207* | *[M+H]+* | *Phenmetrazine* | *59* | *neg_2353* | *1.299* | *441.08719* | *[M-H]-* | *Mutaxanthene A* |
| *9* | *neg_2241* | *1.254* | *425.1012* | *[M-H]-* |  | *60* | *pos_2546* | *0.978* | *372.14813* | *[M+H]+* | *C00051224* |
| *10* | *neg_1481* | *1.406* | *328.11151* | *[M-H]-* |  | *61* | *pos_57* | *3.306* | *109.02712* | *[M+H-H2O]+* | *Public MS/MS: No Annotation* |
| *11* | *neg_1580* | *4.588* | *340.0361* | *[M-H]-* | *methyl 3-methyl-5-(thiophene-2-sulfonyl)-3H,4H,5H,6H,7H-imidazo[4,5-c]pyridine-6-carboxylate* |  |  |  |  |  | *SWMD: 5-(hydroxymethyl)furan-2-carbaldehyde* |
| *12* | *neg_2436* | *1.167* | *452.06235* | *[M-H]-* | *7-chloro-4'-[(1,1-dioxo-2,3-dihydro-1??-thiophen-3-yl)amino]-4,6-dimethoxy-6'-methyl-3H-spiro[1-benzofuran-2,1'-cyclohexan]-3'-ene-2',3-dione* | *62* | *pos_2368* | *1.299* | *351.1058* | *[M+H]+* | *Penicillin V* |
| *13* | *pos_1856* | *3.137* | *293.11304* | *[M+H]+* | *Brachystemidine D* | *63* | *neg_3903* | *6.814* | *1193.08899* | *[M-H]-* |  |
| *14* | *pos_1264* | *5.12* | *239.11717* | *[M+H]+* | *Antibiotic OM 674* | *64* | *pos_2594* | *3.857* | *380.19* | *[M+H]+* | *Fumitremorgin C* |
| *15* | *neg_2388* | *1.299* | *446.02267* | *[M-H2O-H]-* | *1-(4-iodophenyl)-2-{4-methoxy-6-methyl-2H,5H,6H,7H,8H-[1,3]dioxolo[4,5-g]isoquinolin-5-yl}ethan-1-one* | *65* | *neg_1999* | *1.273* | *390.12082* | *[M-H]-* | *Hypogeamicin D* |
| *16* | *neg_2474* | *1.266* | *457.08789* | *[M-H]-* | *7-chloro-4,6-dimethoxy-6'-methyl-4'-[(3-nitrophenyl)amino]-3H-spiro[1-benzofuran-2,1'-cyclohexan]-3'-ene-2',3-dione* | *66* | *pos_1114* | *1.189* | *226.15364* | *[M+H]+* | *UNPD25437* |
| *17* | *pos_848* | *1.173* | *201.08826* | *[M+H]+* |  | *67* | *neg_2473* | *1.133* | *457.07153* | *[2M+FA-H]-* | *(R)-lipoic acid* |
| *18* | *neg_1759* | *4.847* | *359.0383* | *[M-H]-* | *2-(2-{2-[(2-oxo-2H-chromen-7-yl)oxy]acetamido}-1,3-thiazol-4-yl)acetic acid* | *68* | *pos_2116* | *1.477* | *320.14221* | *[M+H]+* | *UNPD32795* |
| *19* | *neg_550* | *1.388* | *211.0714* | *[M-H]-* | *Benzyl Benzoate* | *69* | *pos_3175* | *1.056* | *493.21494* | *[M+H]+* | *UNPD6676* |
| *20* | *pos_1644* | *2.85* | *274.1759* | *[M+H]+* | *UNPD108654* | *70* | *pos_2899* | *1.332* | *430.19229* | *[M+H]+* | *methyl 3-(2,3-dihydro-1,4-benzodioxin-6-yl)-3-{3-hydroxy-4-oxo-6-[(piperidin-1-yl)methyl]-4H-pyran-2-yl}propanoate* |
| *21* | *neg_2953* | *14.886* | *533.24902* | *[M+FA-H]-* | *TMC 89A* | *71* | *pos_2209* | *2.91* | *331.19922* | *[M+H]+* | *Alstonoxine B* |
| *22* | *pos_2564* | *1.416* | *375.19217* | *[M+H]+* | *3-(3,4-dimethylphenoxy)-4,5-dihydroxy-N-(2-oxopiperidin-3-yl)cyclohex-1-ene-1-carboxamide* | *72* | *pos_2428* | *2.462* | *357.21249* | *[M+H]+* | *Leu-Gln-Pro* |
| *23* | *pos_2317* | *2.515* | *343.19812* | *[M+H]+* |  | *73* | *pos_3077* | *3.16* | *466.2305* | *[M+H]+* |  |
| *24* | *pos_969* | *4.322* | *213.06059* | *[M+H]+* | *5-(2-hydroxy-6-oxocyclohex-1-en-1-yl)-3-methyl-1,2??,3-thiadiazolidin-3-ium-5-yl* | *74* | *neg_2739* | *1.477* | *495.05707* | *[M-H]-* | *Public MS/MS: UNPD147636* |
| *25* | *neg_1358* | *5.551* | *314.11481* | *[M-H]-* | *Maremycin D1* |  |  |  |  |  | *SWMD: 7-Phloroeckol* |
| *26* | *neg_2274* | *1.067* | *429.12247* | *[M-H]-* | *Ononin* | *75* | *neg_927* | *3.226* | *263.01843* | *[M-H]-* | *CNP0441445* |
| *27* | *neg_2188* | *1.253* | *417.12198* | *[M-H]-* | *Aloin* | *76* | *pos_513* | *3.237* | *165.07507* | *[M+H]+* | *L-Rhamnulose* |
| *28* | *pos_2208* | *2.263* | *331.1973* | *[M+H]+* | *Momilactone B* | *77* | *pos_3111* | *8.862* | *479.06314* | *[M+H]+* | *Fucofuroeckol B* |
| *29* | *neg_1874* | *1.034* | *375.03516* | *[M-H]-* | *4-bromo-2-[5-(3,4-dimethoxyphenyl)-4,5-dihydro-1H-pyrazol-3-yl]phenol* | *78* | *neg_866* | *1.388* | *255.04948* | *[M-H]-* | *UNPD76400* |
| *30* | *pos_1447* | *1.416* | *254.16347* | *[M+H]+* | *CI 4* | *79* | *neg_1839* | *0.967* | *370.13486* | *[M-H]-* | *Isradipine* |
| *31* | *pos_2170* | *1.322* | *327.12985* | *[M+H]+* | *6,7-Dimethyl-8-(1-D-ribityl)lumazine* | *80* | *pos_3332* | *8.441* | *602.07037* | *[M+H]+* |  |
| *32* | *neg_3176* | *1.045* | *589.16907* | *[M+Na-2H]-* | *Shazhiside methyl ester gentiobioside* | *81* | *pos_2854* | *0.834* | *423.05206* | *[M+H]+* | *Antibiotic C 19393S2* |
| *33* | *neg_1873* | *1.355* | *375.03302* | *[M-H]-* | *2-{[6-(4-chlorobenzenesulfonamido)-hexahydrofuro[3,2-b]furan-3-yl]oxy}acetamide* | *82* | *neg_3217* | *5.236* | *601.05524* | *[M-H]-* |  |
| *34* | *pos_1142* | *1.477* | *228.13245* | *[M+H]+* | *Cordycedipeptide A* | *83* | *pos_1615* | *0.934* | *272.15979* | *[M+H]+* | *Vazabitide A* |
| *35* | *neg_1984* | *1.233* | *388.04407* | *[M-H]-* | *Progoitrin* | *84* | *neg_2653* | *1.333* | *483.10025* | *[M-H]-* | *7-chloro-4'-(4-methanesulfonylpiperazin-1-yl)-4,6-dimethoxy-6'-methyl-3H-spiro[1-benzofuran-2,1'-cyclohexan]-3'-ene-2',3-dione* |
| *36* | *neg_1264* | *0.901* | *305.01572* | *[M-H]-* | *Uridine 2',3'-cyclic phosphate* | *85* | *neg_3391* | *5.961* | *683.03882* | *[M-H]-* |  |
| *37* | *pos_2071* | *0.98* | *315.13* | *[M+NH4]+* |  | *86* | *neg_310* | *4.721* | *177.01717* | *[M-H]-* | *Caffeic acid quinone* |
| *38* | *neg_1521* | *1* | *332.09045* | *[M-H]-* | *Dihydrosanguinarine* | *87* | *pos_1467* | *1.233* | *256.06219* | *[M+H]+* | *2-(4-hydroxy-3-methoxyphenyl)-1,3-thiazolidine-4-carboxylic acid* |
| *39* | *pos_2073* | *0.879* | *315.16232* | *[M+H]+* | *Clomipramine* | *88* | *pos_2100* | *1.056* | *318.12842* | *[M+H]+* | *Clivonine* |
| *40* | *pos_1810* | *2.396* | *288.19849* | *[M+H]+* | *UNPD184215* | *89* | *neg_1962* | *1.326* | *385.1337* | *[M-H]-* | *Brevipolide F* |
| *41* | *pos_2539* | *4.322* | *371.19427* | *[M+H]+* | *18-Hydroxyepialloyohimbine* | *90* | *neg_3696* | *9.549* | *909.40692* | *[M-2H]2-* |  |
| *42* | *neg_2084* | *5.352* | *403.02539* | *[M+FA-H]-* |  | *91* | *neg_3072* | *1.133* | *561.05011* | *[M-H]-* | *TDP-beta-L-rhamnose(2-)* |
| *43* | *pos_2818* | *4.156* | *416.25104* | *[M+H]+* | *N-[[(2R,3R)-5-[(2R)-1-hydroxypropan-2-yl]-3-methyl-8-(3-methylbut-1-ynyl)-6-oxo-3,4-dihydro-2H-pyrido[2,3-b][1,5]oxazocin-2-yl]methyl]-N-methylpropanamide* | *92* | *neg_3542* | *1.787* | *782.34814* | *[M-H]-* | *Auramycin G* |
| *44* | *neg_3295* | *3.702* | *633.05396* | *[M-H]-* |  | *93* | *pos_807* | *3.724* | *197.12634* | *[M+H]+* | *Amiclenomycin* |
| *45* | *neg_358* | *1.499* | *183.02904* | *[M-H]-* | *CNP0431596* | *94* | *neg_1872* | *2.396* | *375.03284* | *[M-H]-* |  |
| *46* | *neg_3292* | *7.312* | *631.04059* | *[M-H]-* | *Secoemestrin D* | *95* | *neg_2676* | *2.617* | *486.11478* | *[M-H]-* | *Renilla luciferyl sulfate* |
| *47* | *pos_2603* | *1.122* | *381.14044* | *[M+H]+* | *Glabrachromene I* | *96* | *neg_868* | *0.879* | *255.10802* | *[M-H]-* | *4-[(E)-2-(3,5-dimethoxyphenyl)ethenyl]phenol* |
| *48* | *neg_2207* | *0.934* | *421.03397* | *[M-H]-* | *Antibiotic C 19393S2* | *97* | *neg_3228* | *6.814* | *607.03204* | *[M-2H]2-* |  |
| *49* | *pos_3333* | *8.43* | *603.08026* | *[M+H]+* | *UNPD61901* | *98* | *pos_2079* | *5.518* | *316.13071* | *[M+H]+* | *Maremycin D1* |
| *50* | *pos_1797* | *4.754* | *287.17206* | *[M+H]+* | *Public MS/MS: N-Methyl-4-dimethylallyltryptophan* | *99* | *neg_3291* | *6.548* | *631.03406* | *[M-H]-* | *Secoemestrin D* |
|  |  |  |  |  | *SWMD: (E)-5-(6-methoxy-2,8-dimethyl-2H-chromen-2-yl)-2-methylpent-2-enal* | *100* | *pos_2412* | *1.299* | *355.16125* | *[M+H]+* | *UNPD90192* |
| *51* | *neg_2007* | *1.286* | *391.02597* | *[M-H]-* | *Antibiotic MM 13902* |  |  |  |  |  |  |

*Library hits occurred in both annotation processes (public MS/MS as well as SWMD) are highlighted in the specific feature annotation (“Public MS/MS” and “SWMD”). SWMD hits include manually supplemented compounds (Supplementary Table 3)

Features are annotated using public MS/MS libraries and a seaweed specific library.
